# Pharmacologic Interventions for Breathlessness in Patients With Advanced Cancer: A Systematic Review and Meta-analysis

**DOI:** 10.1001/jamanetworkopen.2020.37632

**Published:** 2021-02-25

**Authors:** Josephine L. Feliciano, Julie M. Waldfogel, Ritu Sharma, Allen Zhang, Arjun Gupta, Ramy Sedhom, Jeff Day, Eric B. Bass, Sydney M. Dy

**Affiliations:** 1 Sidney Kimmel Comprehensive Cancer Center, Johns Hopkins University, Baltimore, Maryland; 2 Department of Pharmacy, The Johns Hopkins Hospital, Baltimore, Maryland; 3 Department of Health Policy and Management, Johns Hopkins Bloomberg School of Public Health, Baltimore, Maryland; 4 Department of Art as Applied to Medicine, Johns Hopkins University School of Medicine, Baltimore, Maryland; 5 Division of General Internal Medicine, Johns Hopkins University School of Medicine, Baltimore, Maryland

## Abstract

**Importance:**

Improved survival in patients with advanced cancer has increased the need for better understanding of how to manage common symptoms that they may experience, such as breathlessness.

**Objective:**

To assess the benefits and harms associated with pharmacologic interventions for breathlessness in adults with advanced cancer.

**Data Sources:**

PubMed, Embase, CINAHL, Web of Science, and the Cochrane Central Register of Controlled Trials were searched for studies published from database inception through May 31, 2020, using predefined eligibility criteria within a PICOTS (population, intervention, comparator, outcome, timing, setting) format.

**Study Selection:**

Randomized clinical trials (RCTs), non-RCTs, and observational studies with a comparison group that evaluated benefits and/or harms and cohort studies that reported harms were selected.

**Data Extraction and Synthesis:**

Two reviewers independently screened studies for eligibility, serially abstracted data, independently assessed risk of bias, and graded strength of evidence (SOE).

**Main Outcomes and Measures:**

Benefits and harms of pharmacologic interventions were compared, focusing on breathlessness, anxiety, exercise capacity, and health-related quality of life. When possible, meta-analyses were conducted and standardized mean differences (SMDs) calculated.

**Results:**

In this systematic review and meta-analysis, a total of 7729 unique citations were identified, of which 19 studies (17 RCTs and 2 retrospective studies) that included a total of 1424 patients assessed the benefits of medications for management of breathlessness in advanced cancer or reported harms. The most commonly reported type of cancer was lung cancer. Opioids were not associated with more effectiveness than placebo for improving breathlessness (SMD, −0.14; 95% CI, −0.47 to 0.18) or exercise capacity ( SMD, 0.06; 95% CI, −0.43 to 0.55) (SOE, moderate); most studies examined exertional breathlessness. Specific dose and/or route of administration of opioids did not differ in effectiveness for breathlessness (SMD, 0.15; 95% CI, −0.22 to 0.52) (SOE, low). Anxiolytics were not associated with more effectiveness than placebo for breathlessness or anxiety (reported mean between-group difference, −0.52; 95% CI, −1.045 to 0.005) (SOE, low). Evidence for other pharmacologic interventions was limited. Pharmacologic interventions demonstrated some harms compared with usual care, but dropout attributable to adverse events was minimal in these short-term studies (range 3.2%-16%).

**Conclusions and Relevance:**

Evidence did not support the association of opioids or other pharmacologic interventions with improved breathlessness. Given that studies had many limitations, pharmacologic interventions should be considered in selected patients but need to be considered in the context of potential harms and evidence of an association of nonpharmacologic interventions with improved breathlessness.

## Key Points

**Question:**

What are the benefits and harms associated with pharmacologic interventions for managing breathlessness in adults with advanced cancer?

**Findings:**

This systematic review and meta-analysis of 19 studies (17 randomized clinical trials and 2 retrospective studies) found that opioids and anxiolytics were not associated with improved breathlessness in patients with advanced cancer within the limits of the identified studies, which mainly focused on exertional breathlessness for opioids. Data on harms were too limited to draw conclusions.

**Meaning:**

Although the existing data on opioids and pharmacologic interventions do not show an association with improved breathlessness in advanced cancer, they may be considered in selected patients in the context of potential harms and the evidence of an association of nonpharmacologic interventions with improved breathlessness.

## Teaser

This systematic review assesses the benefits and harms of pharmacologic interventions for breathlessness in adults with advanced cancer.

## Introduction

In 2020, the American Cancer Society reported the largest 1-year decrease in cancer deaths, in large part because of emerging therapeutic developments that prolong time living with advanced cancer.^1^ These improvements in survival necessitate evidence on how to improve quality of life (QOL) and symptoms, such as dyspnea, among patients with advanced cancer.

Dyspnea, defined as the sensation of breathlessness, is frequent and debilitating in patients with advanced cancer,^2^ reducing QOL, functional status, and the ability to participate in desired activities.^3^ Oncology guidelines and reviews endorse the use of opioids and anxiolytics as interventions for symptomatic relief of breathlessness in patients with advanced cancer.^4,5,6^ However, these recommendations are driven by older data extrapolated from broad patient populations and from studies that excluded patients with cancer. Patients with cancer may respond differently to these agents because of their unique pathophysiologic mechanisms of breathlessness, concurrent opioid use for pain, and coexisting symptoms. Furthermore, the causes and management of breathlessness may differ at various phases of cancer and may depend on other factors, such as comorbidity, current treatments, and the patient’s life expectancy.

Since the release of previous guidelines and reviews,^4,5,6^ a number of studies^7,8^ have examined the efficacy of therapies for breathlessness in patients who have advanced cancer, and a recent large trial^7^ of specific opioid approaches in mixed populations with chronic breathlessness also did not find a benefit for opioids. Therefore, we conducted a systematic review and meta-analysis to examine the associations of pharmacologic options with improved breathlessness, anxiety, and physiologic outcomes in patients with advanced cancer.

## Methods

This report comes from a broader systematic review that assesses nonpharmacologic and pharmacologic interventions. The full evidence report^9^ has additional details on the methods and other results. With input from a technical expert panel and representatives from the Agency for Healthcare Research and Quality (AHRQ), the American Society for Clinical Oncology, and the Patient-Centered Outcomes Research Institute, we developed a protocol that was posted on the AHRQ Effective Health Care Program’s website. We followed the AHRQ’s Methods Guide for Effectiveness and Comparative Effectiveness Reviews.^10^ This study followed the Preferred Reporting Items for Systematic Review and Meta-analyses (PRISMA) reporting guideline.^11^

### Study Selection

We searched PubMed, Embase, CINAHL, Web of Science, and the Cochrane Central Register of Controlled Trials for articles published from database inception through May 31, 2020, using predefined eligibility criteria within a PICOTS (population, intervention, comparator, outcome, timing, setting) format. We included randomized clinical trials (RCTs), non-RCTs, and observational trials with a concurrent comparison group that enrolled adult patients with advanced cancer and assessed benefits and/or harms of pharmacologic interventions with the intent to alleviate breathlessness. We defined advanced cancer as cancer unlikely to be cured with treatment. We excluded studies with fewer than 10 patients enrolled in each arm and studies with mixed populations in which the percentage of patients with cancer was less than 50%. Key outcomes included in the review are breathlessness, anxiety, exercise capacity, health-related QOL (HRQOL), and physiologic outcomes (see eFigure 1 in the Supplement).

Two reviewers (J.L.F. and J.M.W.) independently screened titles, abstracts, and full text for inclusion. Differences between investigators were resolved through consensus adjudication. We used DistillerSR (Evidence Partners) to manage this process.

### Risk of Bias and Strength of Evidence 

Two reviewers (J.L.F. and J.M.W.) independently assessed the risk of bias (ROB) in studies using the Cochrane Risk of Bias Tool, version 2, for assessing the ROB of RCTs and the Cochrane Risk of Bias Assessment Tool for Non-Randomized Studies of Interventions tool. We graded the strength of evidence (SOE) using the grading scheme recommended by the Methods Guide for Effectiveness and Comparative Effectiveness Reviews.^10^ We applied evidence grades to the bodies of evidence about each comparison for the outcomes we classified during protocol development as the critical outcomes. We assessed the limitations to individual study quality (using individual ROB assessments), consistency, directness, precision, and reporting bias. We classified SOE into 4 grades: high, moderate, low, and insufficient (eTable 1 in the Supplement).

### Statistical Analysis

We used standardized forms in Excel for data extraction (Microsoft Corp). Reviewers (J.L.F. and J.M.W.) extracted information on general study characteristics, participant characteristics, eligibility criteria, interventions, outcome measures, method of ascertainment, and results of each outcome, including measures of variability. One reviewer (J.L.F.) completed data abstraction, and another reviewer (J.M.W.) confirmed the first reviewer’s abstraction for completeness and accuracy.

We conducted meta-analyses for outcomes using a random-effects model with the DerSimonian and Laird method when there were at least 2 sufficiently homogeneous studies. Patient-reported outcomes and clinical scales were standardized by estimating the standardized mean difference (SMD) using the Cohen *d* method. For studies that did not include variability measures, the SD of change in mean was calculated using a correlation coefficient of 0.5, in accordance with methods provided in Fu et al.^12^ We used Cohen classification to categorize effect sizes as small, medium, or large.^13^ When studies reported harms categorically, we calculated relative risks (RRs).

We considered a 10-mm difference on a 100-mm visual analog scale as clinically significant for breathlessness, which corresponds to an SMD of 0.35.^14^ We used Stata statistical software, version 14 (StataCorp LLC) for all meta-analyses. We qualitatively summarized studies that were not amenable to pooling (eAppendix in the Supplement)

## Results

A total of 7729 unique citations were identified, of which 18 studies (17 RCTs and 2 retrospective studies) that included a total of 1424 patients assessed the benefits of medications for management of breathlessness in advanced cancer or reported harms^15,16,17,18,19,20,21,22,23,24,25,26,27,28,29,30,31,32^ (eFigure 2 in the Supplement). The most commonly reported type of cancer was lung cancer. The number of participants in RCTs ranged from 10 to 432; the studies were published between 1993 and 2020. Nine RCTs were placebo controlled, and 8 RCTs and 2 retrospective studies compared results between drugs. Follow-up ranged from 1 minute to 28 days (eTable 2 in the Supplement).

### Breathlessness

Table 1 summarizes the reported effects of the pharmacologic interventions in the 18 studies on breathlessness.^12,13,14,15,19,20,21,22,23,24,25,26,27,28,29,30,31,32^ Detailed information on ROB and SOE are presented in eTables 6 and 7 in the Supplement.

**Table 1.** Summary of Key Results for the Effects of Pharmacologic Interventions on Breathlessness in Patients With Advanced Cancer

Comparison	Evidence of difference	Strength of evidence^a^	No. of studies (No. analyzed)	Key findings	Conclusion
Placebo-controlled comparisons					
Opioids vs placebo	Equivalence	Moderate	6 RCTs (N = 107); fentanyl vs placebo (4),^16,17,18,19^ hydromorphone (nebulized) vs hydromorphone (oral or subcutaneous) vs placebo (nebulized) (1),^15^ morphine vs placebo (1)^20^	Pooled analysis with Charles et al^15^; saline vs nebulized hydromorphone comparison: SMD, −0.12; 95% CI, −0.45 to 0.20; *I*^2^ = 0.0%, *P* = .43; pooled analysis with Charles et al^15^: saline vs systemic hydromorphone comparison: SMD, −0.14; 95% CI: −0.47 to 0.18; *I*^2^ = 0.0%, *P* = .49	Opioids were not more effective than placebo within the limits of the identified studies
Anxiolytics vs placebo	Equivalence	Low	2 RCTs (N = 311); buspirone vs placebo (1),^21^ midazolam vs placebo (1)^31^	Buspirone vs placebo: reported MBGD, −0.52; 95% CI, −1.045 to 0.005; midazolam vs placebo: no statistically significant difference between groups (*P* = .75) at 60 min; unable to calculate SMD, data presented as number of spray bottles rather than number of participants	Anxiolytics were no more effective than placebo
Corticosteroids vs placebo	No conclusion drawn	Insufficient	1 RCT (N = 28); dexamethasone vs placebo (1)^22^	Calculated SMD, −0.06; 95% CI, −0.70 to 0.58	NA
Drug-drug comparisons					
Opioids vs opioids	Equivalence	Low	7 RCTs (N = 132)^15,23,24,27,28,30,32^; subcutaneous vs sublingual morphine (1), subcutaneous vs nebulized morphine (1), high- vs low-dose sublingual fentanyl (1), low- vs high-dose opioids (drug unspecified) (1), hydromorphone (nebulized) vs hydromorphone (oral or subcutaneous) vs placebo (nebulized) (1), buccal fentanyl vs oral morphine (1), oral morphine hydrochloride vs oral morphine sulfate (1)	Pooled analysis: SMD, 0.15; 95% CI, −0.22 to 0.52; *I*^2^ = 4.8%, *P* = .37	No difference in effectiveness between opioid doses or routes in improving breathlessness
Opioids vs anxiolytics	Equivalence	Low	2 RCTs (N = 108)^25,26^; oral morphine vs oral midazolam (1), subcutaneous morphine vs subcutaneous midazolam vs combination (1)	For breathlessness intensity: 1 study found midazolam was more effective than morphine at 5 d (*P* < .001); another study found no significant differences between groups at 24 or 48 h; for categorical variable of percentage not experiencing breathlessness relief: calculated RR, 0.075; 95% CI, 0.004 to 1.270; calculated RR, 1.33; 95% CI, 1.02 to 1.75	Opioids were not more effective than anxiolytics for improving breathlessness
Opioids vs corticosteroids vs bronchodilators	No conclusion drawn	Insufficient	1 Retrospective cohort (N = 343)^29^; morphine vs methylprednisolone vs aminophylline (1)	Methylprednisolone vs aminophylline: calculated SMD, 0.41; 95% CI, 0.15 to 0.68; morphine vs aminophylline: calculated SMD, 1.18; 95% CI, 0.90 to 1.46; morphine vs methylprednisolone: calculated SMD, 0.76; 95% CI, 0.49 to 1.03	NA

Abbreviations: MBGD, mean between-group difference; NA, not applicable; RCT, randomized clinical trial; RR, relative risk; SMD, standardized mean difference.

^a^ Moderate strength indicates that further research may change the result; low strength indicates low confidence that the evidence reflects the true effect, and further research is very likely to change the result; and insufficient evidence indicates that evidence is unavailable or does not permit a conclusion.

#### Opioids vs Placebo

**Figure 1.**
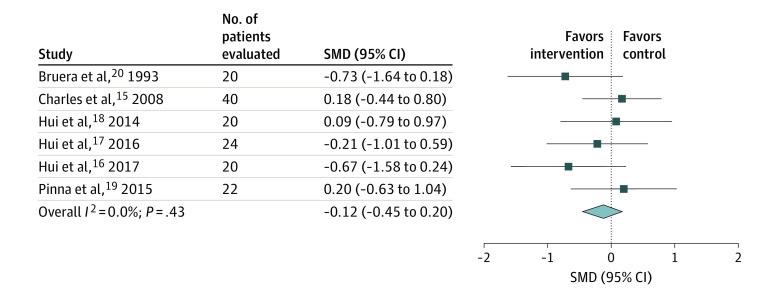
Meta-analysis of the Effects on Breathlessness in Randomized Clinical Trials Comparing Opioids With Placebo in Patients With Advanced Cancer (Including the Charles et al^15^ Saline vs Nebulized Hydromorphone Comparison) SMD indicates standardized mean difference. The squares indicate the SMDs, the horizontal lines the 95% CIs, and the diamond the overall result.

**Figure 2.**
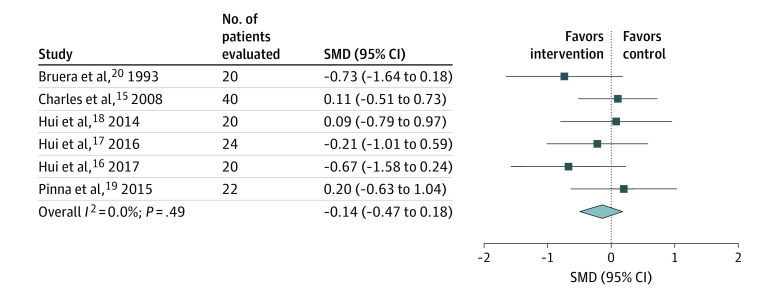
Meta-analysis of the Effects on Breathlessness in Randomized Clinical Trials Comparing Opioids With Placebo in Patients With Advanced Cancer (Including the Charles et al^15^ Saline vs Systemic Hydromorphone Comparison) SMD indicates standardized mean difference. The squares indicate the SMDs, the horizontal lines the 95% CIs, and the diamond the overall result.

Six RCTs^15,16,17,18,19,20^ assessed the effect of opioids compared with placebo on breathlessness, and most of these studies examined exertional breathlessness. Four RCTs^16,17,18,19^ compared fentanyl products, 1 RCT^15^ evaluated hydromorphone, and 1 RCT^20^ evaluated subcutaneous morphine. We performed 2 meta-analyses to reflect the 2 placebo comparisons provided in 1 of the RCTs and equivalence with and without the nebulized arm^15^ (calculated SMD for saline vs nebulized hydromorphone comparison, −0.12; 95% CI, −0.45 to 0.20; *I*^2^ = 0.0%) (calculated SMD for saline vs systemic hydromorphone comparison, −0.14; 95% CI, −0.47 to 0.18; *I*^2^ = 0.0%) (Figure 1^15,16,17,18,19,20^ and Figure 2^15,16,17,18,19,20^). All studies reported an active placebo effect on within-group differences. On the basis of the overall pooled results from the meta-analysis, opioids were not more effective than placebo for improving breathlessness in patients with advanced cancer (SOE, moderate).

#### Anxiolytics vs Placebo

Two RCTs^21,31^ assessed the effect of anxiolytics compared with placebo. One study,^21^ evaluating buspirone vs placebo, found no statistically significant difference between groups (reported mean between-group difference, −0.52; 95% CI, −1.045 to 0.005). The other RCT^31^ of intranasal midazolam vs placebo also found no statistically significant difference in breathlessness between groups. On the basis of the available evidence, anxiolytics were not more effective than placebo for the treatment of breathlessness (SOE, low). We did not perform a meta-analysis given the different mechanisms of actions for these anxiolytics.

#### Corticosteroids vs Placebo

One RCT^22^ of oral dexamethasone compared with placebo found no statistically significant effect in breathlessness (calculated SMD, −0.06; 95% CI, −0.70 to 0.58). Evidence was insufficient to evaluate the effectiveness of corticosteroids vs placebo for the treatment of breathlessness.

#### Opioids vs Opioids

**Figure 3.**
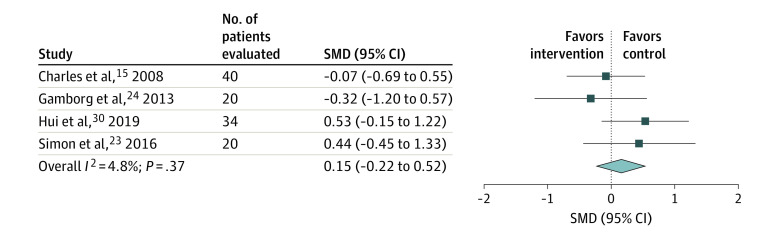
Meta-analysis of the Effects on Breathlessness in Randomized Clinical Trials Comparing Opioids With Opioids in Patients With Advanced Cancer SMD indicates standardized mean difference. The squares indicate the SMDs, the horizontal lines the 95% CIs, and the diamond the overall result.

Seven RCTs^15,23,24,27,28,30,32^ compared the effect of different routes of administration or different doses of opioids for treatment of breathlessness in patients with advanced cancer. Meta-analysis of 4 of the RCTs^15,23,24,30^ found no difference between opioid doses or routes in treating breathlessness in patients with advanced cancer (calculated SMD, 0.15; 95% CI, −0.22 to 0.52; *I*^2^ = 4.8%) (Figure 3). Three of the RCTs^27,28,32^ were not included in the analysis because they reported results as median rather than mean, data were derived from figures, or not enough information was included for calculations. Two of these RCTs^27,28^ reported no statistically significant differences between groups, and 1 RCT^32^ reported a significant difference favoring morphine. We concluded that there were no differences between opioid doses or routes.

#### Opioids vs Anxiolytics

Two RCTs^25,26^ evaluated the effect of midazolam compared with morphine or the combination of both drugs. One RCT^25^ found that midazolam was more effective at relieving breathlessness at 5 days, and another RCT^26^ found no statistically significant differences in breathlessness intensity at 24 or 48 hours. The combination morphine and midazolam group, however, had a statistically significantly higher percentage of patients reporting breathlessness relief than either agent alone at 24 hours, which was persistent compared with the midazolam-alone group at 48 hours. We concluded that opioids were not more effective than anxiolytics (midazolam) for improving breathlessness (SOE, low). Given the heterogeneity of patient populations, we did not conduct a meta-analysis.

#### Opioids vs Corticosteroids vs Bronchodilators

One single-center retrospective cohort study^29^ evaluated morphine compared with methylprednisolone or aminophylline. Specifically, methylprednisolone improved breathlessness more than aminophylline (calculated SMD, 0.41; 95% CI, 0.15-0.68), and morphine improved breathlessness more than either of the other agents (morphine vs aminophylline: calculated SMD, 1.18; 95% CI, 0.90-1.46; morphine vs methylprednisolone: calculated SMD, 0.76; 95% CI, 0.49-1.03). Given only 1 retrospective cohort study,^29^ we concluded that evidence was insufficient to evaluate the effectiveness of opioids vs corticosteroids vs bronchodilators for treatment of breathlessness.

### Anxiety, Exercise Capacity, and HRQOL

Table 2 summarizes the effects of the pharmacologic interventions on anxiety, exercise capacity, and HRQOL. Detailed information on ROB and SOE are presented in eTables 6 and 7 in the Supplement. Only placebo-controlled comparisons are reported for these outcomes. Two RCTs^21,31^ of anxiolytics compared with placebo reported no difference in the treatment of anxiety in patients with advanced cancer. We concluded that anxiolytics were no more effective than placebo in treating anxiety (SOE, low). One RCT^22^ of oral dexamethasone compared with placebo in patients with advanced cancer found no difference on HRQOL using the European Organization for Research and Treatment of Cancer Quality of Life Questionnaire–Core Questionnaire. Three RCTs^17,18,19^ of fentanyl compared with placebo reported results in terms of distance in a 6-minute walk test. Meta-analysis of 3 RCTs^16,17,18^ found no differences in 6-minute walk distance (calculated SMD, 0.06; 95% CI, −0.43 to 0.55; *I*^2^  = 0.0%) (eFigure 3 in the Supplement). We concluded that opioids were not more effective than placebo for improving exercise capacity (SOE, moderate).

**Table 2.** Summary of Key Results for the Effects of Pharmacologic Interventions on Anxiety, Health-Related Quality of Life, and Exercise Capacity in Patients With Advanced Cancer

Comparison	Evidence of difference	Strength of evidence^a^	No. of studies (No. analyzed)	Key findings	Conclusion
					
Anxiolytics vs placebo for anxiety	Equivalence	Low	2 RCTs (N = 311)^21,31^; buspirone vs placebo (1); intranasal midazolam vs placebo	Buspirone vs placebo: no statistically significant differences between groups; unable to calculate SMD; intranasal midazolam vs placebo: no difference between arms reported by authors	Anxiolytics were not more effective than placebo for improving anxiety
					
Corticosteroids vs placebo for health-related quality of life	No conclusion drawn	Insufficient	1 RCT (N = 28)^22^; oral dexamethasone vs placebo	Calculated SMD, −0.06; 95% CI, −0.70 to 0.58	NA
					
Opioids vs placebo for exercise capacity	Equivalence	Moderate	3 RCTs (N = 77)^17,18,19^; fentanyl vs placebo	Pooled analysis of 3 studies: SMD, 0.06; 95% CI, −0.43 to 0.55; *I*^2^ = 0.0%, *P* = .90; fourth study reported no significant differences between groups; unable to calculate SMD, data reported as medium rather than mean	Opioids were not more effective than placebo for improving exercise capacity

Abbreviations: NA, not applicable; RCT, randomized clinical trial; SMD, standardized mean difference.

^a^ Moderate strength indicates that further research may change the result; low strength indicates low confidence that the evidence reflects the true effect, and further research is very likely to change the result; and insufficient evidence indicates that evidence is unavailable or does not permit a conclusion.

### Physiologic Outcomes

eTable 3 in the Supplement summarizes the physiologic outcomes in 12 studies. ^15,17,18,19,20,23,24,25,26,27,28,29,30^ No study reported any significant effects from any drugs on physiologic outcomes (eFigures 4–13 in the Supplement).

### Harms and Dropout

Fourteen studies^15,16,17,18,19,21,22,25,26,27,29,30,31,33^ (12 RCTs^15,16,17,18,19,21,22,25,26,27,30,31^ and 2 retrospective studies^29,33^) addressed the adverse effects and dropout associated with pharmacologic interventions (eTable 4 in the Supplement). No study reported harms of opioids compared with other opioids or other opioid dosing. None of these studies reported on headaches or opioid use disorder.

### Central Nervous System Effects 

Three RCTs^16,17,18^ comparing opioids with placebo assessed central nervous system adverse effects. A meta-analysis found no statistically significant difference between fentanyl and placebo for dizziness (RR, 0.68; 95% CI, 0.15-3.11; *I*^2^ = 0.0%; *P* = .41) (eFigure 14 in the Supplement). A meta-analysis on 2 of the 3 RCTs to evaluate drowsiness found no statistically significant difference between fentanyl or placebo (RR, 0.38; 95% CI, 0.06-2.27; *I*^2^  = 0.0%; *P* = .70) (eFigure 15 in the Supplement). Finally, a meta-analysis on all 3 RCTs^16,17,18^ found no statistically significant difference in fatigue between groups (mean between-group difference: SMD, −0.15; 95% CI, −0.64 to 0.34; *I*^2^ = 0.0%; *P* = .67) (eFigure 16 in the Supplement). For central nervous system adverse effects, corticosteroids had lower rates of drowsiness compared with placebo or opioids, but results for dizziness were inconsistent.

### Gastrointestinal Effects 

For gastrointestinal adverse effects, opioids had higher rates of constipation compared with steroids (RR, 0.01; 95% CI, 0-0.15) for the methylprednisolone and aminophylline groups compared with morphine; studies that compared opioids with placebo were short term and did not report this adverse effect. Study reporting was insufficient to draw conclusions about nausea for any of the pharmacologic comparison groups.

### Dropout

The rate of dropout attributable to adverse effects was reported in 5 studies.^15,16,21,22,25^ Adverse effects led to dropout in a small percentage of patients (range, 3.2%-16%) for all types of pharmacologic interventions (eTable 5 in the Supplement).

## Discussion

This systematic review and meta-analysis found that opioids were not associated with more effectiveness than placebo and not more effective than other pharmacologic interventions for improving breathlessness for patients with advanced cancer. Anxiolytics were not associated with more effectiveness than placebo for these outcomes, with low SOE. Furthermore, there was limited or insufficient evidence that pharmacologic therapies impacted other outcomes, including anxiety, HRQOL, or exercise capacity.

Existing guidelines emphasize the use of opioids and benzodiazepines for management of breathlessness in patients with advanced cancer.^4,5^ The findings of the current study differ somewhat from previously published reviews,^6,34^ and several factors may contribute to these differences. The results of this review must be interpreted within the context of the included RCTs. First, this study includes 19 studies, 10 of which have been published since the Cochrane review and were not included in previous guidelines.^16,17,18,19,21,22,23,29,30,31^ Many of those more recent studies^16,17,19,21,22,23^ are randomized and placebo controlled and evaluate opiates for breathlessness, with most studies demonstrating improvement in the placebo groups. This active placebo effect may have contributed to the lack of benefit over placebo that was found in the current study.

Second, at least half of the studies evaluated in the Cochrane review were in populations other than cancer, whereas this review includes studies in which most patients had advanced cancer. This difference is important because breathlessness in patients with advanced cancer may have unique features and considerations. The patterns and physiologic mechanisms of breathlessness in patients with advanced cancer may be different from those without cancer and can be associated with the cancer itself or with treatments or adverse effects of treatments. Breathlessness in patients with cancer may also be associated with concurrent symptoms, such as anxiety, fatigue, or pain.^35^ Thus, it is important to focus on this patient population.

Third, the included studies differed widely in the patient populations (medical comorbidities, concurrent opioid or other supportive medications, concurrent interventions to relieve, or prognosis), outcomes evaluated, and measurement instruments used. For example, management of breathlessness in patients with a long-term prognosis compared with those at the end stages of advanced cancer may have different contributing factors and interventions for those factors. The heterogeneity limited the ability to synthesize results.

Fourth, accruing patients to these types of studies can be difficult. In clinical practice, it may be challenging to apply study results to real-world clinical practice, where numerous additional factors may be introduced and cannot all be controlled for. Collaborative group efforts and well-designed, real-world evidence data collection and studies may help to address some of these issues in complex patient populations in whom accrual can be challenging or potentially unethical, such as actively dying patients.

Fifth, determining how best to measure outcomes of interest can be challenging. Breathlessness may be measured at different time points and using different methods. The data included in the studies in this meta-analysis were reported before and after intervention, but interpretation outside the studied time points may be limited; longer-term studies are needed. Furthermore, breathlessness is a complex and subjective multidimensional patient experience, but the studies in this review used unidimensional scales. Consensus recommendations prefer multidimensional scales^36^; for example, the Personalized Dyspnea Intensity Goal^37^ can personalize outcome measurements in the context of patients’ expectations and may be more clinically relevant. Multidimensional measures could help improve the evidence of interventions for breathlessness.

In oncology, advanced disease is not synonymous with end of life and can include patients with long life expectancies, particularly in the era of improved survival and new therapeutic agents. For example, a patient with advanced breast cancer, prostate cancer, or lung cancer may have prolonged survival exceeding 5 years, despite having incurable disease. Recommendations for pharmacologic interventions need to be integrated within the context of life expectancy and improved survival for many advanced and incurable cancers.

Implementing the findings of this review may seem counter to previous guidelines and reviews. There is a role for pharmacologic agents, such as opioids and anxiolytics, in the treatment of patients with advanced cancer, but practitioners must account for many factors. Future studies should incorporate a multidimensional common measure of breathlessness. Evaluation of more real-world evidence on the efficacy of pharmacologic agents may help to better explain how and when to implement interventions and how to integrate them with other interventions, such as nonpharmacologic interventions, which have demonstrated evidence of the association with improved breathlessness.^38^ Future studies also need to incorporate life expectancy as the number of patients with advanced cancer who have long survival grows.

### Limitations

This review has limitations, particularly in the context of individual study quality and the intrinsic limitations of the published literature. As previously mentioned, breathlessness is a subjective multidimensional phenomenon that can be difficult to capture with the unidimensional scales in this review. The RCTs reported different types of breathlessness (chronic, episodic, and exertional) in various settings (eg, outpatient clinics and palliative care units) with varied duration of follow-up. In addition, minimum clinically important differences that were defined in our analyses were extrapolated from studies that include broad patient populations, such as patients who have breathlessness from chronic obstructive pulmonary disease; these clinically important differences could be different in advanced cancer. Furthermore, most of the studies were small, highlighting the difficulty accruing patients for these types of studies, and had major limitations on risk of bias assessment, with missing or incomplete information about the randomization data and about outcomes measurements and data (eTable 6 in the Supplement). Treating crossover trial data similarly to parallel studies may have resulted in slightly wider CIs, but a corrected SE was imputed when possible to address this issue. In addition, studies used unidimensional scales for breathlessness rather than recommended multidimensional constructs, and many did not report important outcomes other than breathlessness. Reporting harms and adverse events was inconsistent among the studies, making it difficult to combine results. In addition, some studies that studied interventions aimed at relieving reversible causes of breathlessness, such as pleural effusions or anemia, were excluded, as were studies that targeted closely related symptoms, such as pain or cough.

## Conclusions

This study found that pharmacologic interventions were not associated with more effectiveness than placebo for the management of breathlessness in patients with advanced cancer. Practitioners may need to reassess the role of pharmacologic agents, such as opioids and anxiolytics, in the management of breathlessness for patients with advanced cancer, taking into consideration many patient-related factors.

## Notes

Supplement. **eAppendix.** Methods Details **eTable 1.** Definitions of the Grades of Overall Strength of Evidence **eTable 2.** Characteristics of Included Studies **eTable 3.** Summary of Findings for the Effects of Pharmacological Interventions on Physiologic Outcomes in Patients With Advanced Cancer **eTable 4.** List of Studies Reporting Harms and Dropouts in Studies of Pharmacological Interventions for Breathlessness in Patients With Advanced Cancer **eTable 5.** Rate of Dropouts Due to Adverse Effects of Pharmacological Interventions for Breathlessness in Patients With Advanced Cancer **eTable 6.** Risk of Bias of Randomized Controlled Trials (A) and Observational Studies (B) That Evaluate the Effects of Pharmacologic Interventions **eTable 7.** Strength of Evidence of Studies That Evaluate the Effects of Pharmacologic Interventions **eFigure 1.** Analytic Framework for Evaluating Interventions for Breathlessness in Patients With Advanced Cancer **eFigure 2.** Study Search and Preferred Reporting Items for Systematic Reviews and Meta-analyses Flowchart **eFigure 3.** Meta-analysis of the Effects of Exercise Capacity Measures in Randomized Controlled Trials Comparing Opioids With Placebo In Patients With Advanced Cancer **eFigure 4.** Meta-analysis of the Effects of Placebo vs Opioids on Blood Pressure in Patients With Advanced Cancer in Inpatient Hospice or Palliative Care Units **eFigure 5.** Meta-analysis of the Effects of Placebo vs Opioids on Heart Rate in Patients With Advanced Cancer in Inpatient Hospice or Palliative Care Units (Charles, 2008 et al^15^ Saline vs Nebulized Hydromorphone Comparison) **eFigure 6.** Meta-analysis of the Effects of Placebo vs Opioids on Heart Rate in Patients With Advanced Cancer in Inpatient Hospice or Palliative Care Units (Charles, 2008 et al^15^ Saline vs Systemic Hydromorphone Comparison) **eFigure 7.** Meta-analysis of the Effects of Opioids vs Opioids on Heart Rate in Patients With Advanced Cancer in Inpatient Hospice or Palliative Care Units **eFigure 8.** Meta-analysis of the Effects of Placebo vs Opioids on Oxygen Saturation in Patients With Advanced Cancer in Inpatient Hospice or Palliative Care Units (Charles, 2008 et al^15^ Saline vs Nebulized Hydromorphone Comparison) **eFigure 9.** Meta-analysis of the Effects of Placebo vs Opioids on Oxygen Saturation in Patients With Advanced Cancer in Inpatient Hospice or Palliative Care Units (Charles, 2008 et al^15^ Saline Vs Systemic Hydromorphone Comparison) **eFigure 10.** Meta-analysis of the Effects of Opioids vs Opioids on Oxygen Saturation in Patients With Advanced Cancer in Inpatient Hospice or Palliative Care Units **eFigure 11.** Meta-analysis of the Effects of Placebo vs Opioids on Respiratory Rates in Patients With Advanced Cancer in Inpatient Hospice or Palliative Care Units (Charles, 2008 et al^15^ Saline vs Nebulized Hydromorphone Comparison) **eFigure 12.** Meta-analysis of the Effects of Placebo vs Opioids on Respiratory Rates in Patients With Advanced Cancer in Inpatient Hospice or Palliative Care Units (Charles, 2008 et al^15^ Saline vs Systemic Hydromorphone Comparison) **eFigure 13.** Meta-analysis of the Effects of Opioids vs Opioids on Respiratory Rates in Patients With Advanced Cancer in Inpatient Hospice or Palliative Care Units **eFigure 14.** Meta-analysis of the Effects of Placebo vs Opioids on Dizziness Outcomes in Patients With Advanced Cancer in Inpatient Hospice or Palliative Care Units **eFigure 15.** Meta-analysis of the Effects of Placebo vs Opioids on Drowsiness Outcomes in Patients With Advanced Cancer in Inpatient Hospice or Palliative Care Units **eFigure 16.** Meta-analysis of the Effects of Placebo vs Opioids on Fatigue Outcomes in Patients With Advanced Cancer in Inpatient Hospice or Palliative Care Units
