# Supplementary material for: Pharmacologic Interventions for Breathlessness in Patients With Advanced Cancer: A Systematic Review and Meta-analysis
Source: JAMA Netw Open. 2021 Feb 25;4(2):e2037632. doi: 10.1001/jamanetworkopen.2020.37632 (PMC7907959; doi:10.1001/jamanetworkopen.2020.37632)
Supplement: Supplement. — eAppendix. Methods Details eTable 1. Definitions of the Grades of Overall Strength of Evidence eTable 2. Characteristics of Included Studies eTable 3. Summary of Findings for the Effects of Pharmacological Interventions on Physiologic Outcomes in Patients With Advanced Cancer eTable 4. List of Studies Reporting Harms and Dropouts in Studies of Pharmacological Interventions for Breathlessness in Patients With Advanced Cancer eTable 5. Rate of Dropouts Due to Adverse Effects of Pharmacological Interventions for Breathlessness in Patients With Advanced Cancer eTable 6. Risk of Bias of Randomized Controlled Trials (A) and Observational Studies (B) That Evaluate the Effects of Pharmacologic Interventions eTable 7. Strength of Evidence of Studies That Evaluate the Effects of Pharmacologic Interventions eFigure 1. Analytic Framework for Evaluating Interventions for Breathlessness in Patients With Advanced Cancer eFigure 2. Study Search and Preferred Reporting Items for Systematic Reviews and Meta-analyses Flowchart eFigure 3. Meta-analysis of the Effects of Exercise Capacity Measures in Randomized Controlled Trials Comparing Opioids With Placebo In Patients With Advanced Cancer eFigure 4. Meta-analysis of the Effects of Placebo vs Opioids on Blood Pressure in Patients With Advanced Cancer in Inpatient Hospice or Palliative Care Units eFigure 5. Meta-analysis of the Effects of Placebo vs Opioids on Heart Rate in Patients With Advanced Cancer in Inpatient Hospice or Palliative Care Units (Charles, 2008 et al15 Saline vs Nebulized Hydromorphone Comparison) eFigure 6. Meta-analysis of the Effects of Placebo vs Opioids on Heart Rate in Patients With Advanced Cancer in Inpatient Hospice or Palliative Care Units (Charles, 2008 et al15 Saline vs Systemic Hydromorphone Comparison) eFigure 7. Meta-analysis of the Effects of Opioids vs Opioids on Heart Rate in Patients With Advanced Cancer in Inpatient Hospice or Palliative Care Units eFigure 8. Meta-analysis of the Effects of Placeb [file jamanetwopen-e2037632-s001.pdf]

Supplementary Online Content

Feliciano JL, Waldfogel JM, Sharma R, et al. Pharmacologic interventions for breathlessness in patients with advanced cancer: a systematic review and meta-analysis. *JAMA Netw Open*. 2021;4(2):e2037632. doi:10.1001/jamanetworkopen.2020.37632

eAppendix. Methods Details
eTable 1. Definitions of the Grades of Overall Strength of Evidence
eTable 2. Characteristics of Included Studies
eTable 3. Summary of Findings for the Effects of Pharmacological Interventions on Physiologic Outcomes in Patients With Advanced Cancer
eTable 4. List of Studies Reporting Harms and Dropouts in Studies of Pharmacological Interventions for Breathlessness in Patients With Advanced Cancer
eTable 5. Rate of Dropouts Due to Adverse Effects of Pharmacological Interventions for Breathlessness in Patients With Advanced Cancer
eTable 6. Risk of Bias of Randomized Controlled Trials (A) and Observational Studies (B) That Evaluate the Effects of Pharmacologic Interventions
eTable 7. Strength of Evidence of Studies That Evaluate the Effects of Pharmacologic Interventions
eFigure 1. Analytic Framework for Evaluating Interventions for Breathlessness in Patients wWth Advanced Cancer
eFigure 2. Study Search and Preferred Reporting Items for Systematic Reviews and Meta-analyses Flowchart
eFigure 3. Meta-analysis of the Effects of Exercise Capacity Measures in Randomized Controlled Trials Comparing Opioids With Placebo In Patients With Advanced Cancer
eFigure 4. Meta-analysis of the Effects of Placebo vs Opioids on Blood Pressure in Patients With Advanced Cancer in Inpatient Hospice or Palliative Care Units
eFigure 5. Meta-analysis of the Effects of Placebo vs Opioids on Heart Rate in Patients With Advanced Cancer in Inpatient Hospice or Palliative Care Units (Charles, 2008 et al[15] Saline vs Nebulized Hydromorphone Comparison)
eFigure 6. Meta-analysis of the Effects of Placebo vs Opioids on Heart Rate in Patients With Advanced Cancer in Inpatient Hospice or Palliative Care Units (Charles, 2008 et al[15] Saline vs Systemic Hydromorphone Comparison)
eFigure 7. Meta-analysis of the Effects of Opioids vs Opioids on Heart Rate in Patients With Advanced Cancer in Inpatient Hospice or Palliative Care Units
eFigure 8. Meta-analysis of the Effects of Placebo vs Opioids on Oxygen Saturation in Patients With Advanced Cancer in Inpatient Hospice or Palliative Care Units (Charles, 2008 et al[15] Saline vs Nebulized Hydromorphone Comparison)
eFigure 9. Meta-analysis of the Effects of Placebo vs Opioids on Oxygen Saturation in Patients With Advanced Cancer in Inpatient Hospice or Palliative Care Units (Charles, 2008 et al[15] Saline Vs Systemic Hydromorphone Comparison)
eFigure 10. Meta-analysis of the Effects of Opioids vs Opioids on Oxygen Saturation in Patients With Advanced Cancer in Inpatient Hospice or Palliative Care Units
eFigure 11. Meta-analysis of the Effects of Placebo vs Opioids on Respiratory Rates in Patients With Advanced Cancer in Inpatient Hospice or Palliative Care Units (Charles, 2008 et al[15] Saline vs Nebulized Hydromorphone Comparison)
eFigure 12. Meta-analysis of the Effects of Placebo vs Opioids on Respiratory Rates in Patients With Advanced Cancer in Inpatient Hospice or Palliative Care Units (Charles, 2008 et al[15] Saline vs Systemic Hydromorphone Comparison)
eFigure 13. Meta-analysis of the Effects of Opioids vs Opioids on Respiratory Rates in Patients With Advanced Cancer in Inpatient Hospice or Palliative Care Units
eFigure 14. Meta-analysis of the Effects of Placebo vs Opioids on Dizziness Outcomes in Patients With Advanced Cancer in Inpatient Hospice or Palliative Care Units
eFigure 15. Meta-analysis of the Effects of Placebo vs Opioids on Drowsiness Outcomes in Patients With Advanced Cancer in Inpatient Hospice or Palliative Care Units
eFigure 16. Meta-analysis of the Effects of Placebo vs Opioids on Fatigue Outcomes in Patients With Advanced Cancer in Inpatient Hospice or Palliative Care Units
This supplementary material has been provided by the authors to give readers additional information about their work.

eAppendix. Methods details

Statistical heterogeneity

We evaluated statistical heterogeneity among studies using an I^2 statistic. We considered an I^2 value greater than 50% as substantial statistical heterogeneity. When we found substantial heterogeneity, we evaluated whether quantitative synthesis was appropriate or conducted sensitivity analysis when applicable.

Clinically Important Difference

Although a minimally clinically important difference (MCID) in breathlessness intensity has not been formally established, there are data available to help guide this determination. In heart failure, studies have identified a difference on the VAS between 10 and 21.1 mm as clinically significant[1-3]. Similarly, data for chronic refractory breathlessness and COPD suggest a difference of 10mm on the VAS or 0.8 on the Borg scale as clinically.[4,5] In a cancer population, data from a study of breathlessness from malignant pleural effusion suggest a difference on the VAS of 19mm is clinically significant and a population of advanced cancer patients admitted to a palliative care unit considered a difference on the NRS of 2.1 to be clinically important.[6,7]. Given the available data, we considered a difference on the VAS of 10mm or greater as clinically meaningful.

Cross-over Trials

For cross-over trials, we treated data similarly to parallel studies, using the measurement from the intervention periods and the measurements from the control periods Where possible we used a correlation co-efficient to impute a corrected standard of error, as this method may result in slightly wider confidence intervals.[8]

© 2021 Feliciano JL et al. *JAMA Network Open.*

References

1. Pang PS, Lane KA, Tavares M, et al. Is there a clinically meaningful difference in patient reported dyspnea in acute heart failure? An analysis from URGENT Dyspnea. *Heart & lung : the journal of critical care.* 2017;46(4):300-307.
2. Ander DS, Aisiku IP, Ratcliff JJ, Todd KH, Gotsch K. Measuring the dyspnea of decompensated heart failure with a visual analog scale: how much improvement is meaningful? *Congestive heart failure (Greenwich, Conn).* 2004;10(4):188-191.
3. Oxberry SG, Bland JM, Clark AL, Cleland JGF, Johnson MJ. Minimally clinically important difference in chronic breathlessness: Every little helps. *American Heart Journal.* 2012;164(2):229-235.
4. Johnson MJ, Bland JM, Oxberry SG, Abernethy AP, Currow DC. Clinically important differences in the intensity of chronic refractory breathlessness. *Journal of Pain and Symptom Management.* 2013;46(6):957-963.
5. Oliveira A, Machado A, Marques A. Minimal Important and Detectable Differences of Respiratory Measures in Outpatients with AECOPD(dagger). COPD. 2018 Oct;15(5):479-88. doi: 10.1080/15412555.2018.1537366.
6. Mishra EK, Corcoran JP, Hallifax RJ, Stradling J, Maskell NA, Rahman NM. Defining the minimal important difference for the visual analogue scale assessing dyspnea in patients with malignant pleural effusions. *PloS one.* 2015;10(4):e0123798.
7. Mercadante S, Adile C, Aielli F, et al. Personalized Goal for Dyspnea and Clinical Response in Advanced Cancer Patients. J Pain Symptom Manage. 2019;57(1):79-85.
8. Higgins JPT, Thomas J, Chandler J, Cumpston M, Li T, Page MJ, Welch VA (editors). Chapter 23: Including variants on randomized trials. Cochrane Handbook for Systematic Reviews of Interventions version 6.1 (updated September 2020). Cochrane, 2020.

© 2021 Feliciano JL et al. *JAMA Network Open.*

eTable 1. Definitions of the grades of overall strength of evidence

Grade	Definition
High	We are very confident that the estimate of effect lies close to the true effect for this outcome. The body of evidence has few or no deficiencies. We believe that the findings are stable (i.e., another study would not change the conclusions).
Moderate	We are moderately confident that the estimate of effect lies close to the true effect for this outcome. The body of evidence has some deficiencies. We believe that the findings are likely to be stable, but some doubt remains.
Low	We have limited confidence that the estimate of effect lies close to the true effect for this outcome. The body of evidence has major or numerous deficiencies (or both). We believe that additional evidence is needed before concluding either that the findings are stable or that the estimate of effect is close to the true effect.
Insufficient	We have no evidence, we are unable to estimate an effect, or we have no confidence in the estimate of effect for this outcome. No evidence is available, or the body of evidence has unacceptable deficiencies, precluding reaching a conclusion.

© 2021 Feliciano JL et al. *JAMA Network Open.*

eTable 2. Characteristics of included studies

Author, year<br><br>Study design	Participants, n	Cancer type	Setting	Age	Followup	Breathlessness	Anxiety	Exercise capacity	HRQOL	Physiologic outcomes	Risk of bias
**Placebo vs Anxiolytics**											
Hardy, 2016[31]<br><br>RCT	73	Cancer (not specified)	Hospital, Hospice (inpatient)	Median 70	14 days	X	X				Low
Peoples, 2016[21]<br><br>RCT	379	Lung, Breast, Gastrointestinal, Other	Oncology clinic	62.9-64	28 days	X	X				Some concerns
**Placebo vs Corticosteroids**											
Hui, 2016[22]<br><br>RCT	41	NSCLC, small cell lung cancer, Mesothelioma, other	Oncology, Palliative care clinic	63	14 days	X			X		Low
**Placebo vs Opioids**											
Bruera, 1993[20]<br><br>RCT: Crossover	10	Lung (others not specified)	Not reported	NR	60 minutes	X				X	High
Charles, 2008[15]<br><br>RCT: Crossover	20	Breast, Lung, Mesothelioma, Prostate, Renal	Hospice (home), Hospice (inpatient)	69	60 minutes	X				X	High
Hui, 2014[18]<br><br>RCT	20	Breast, Gastrointestinal, Genitourinary, Gynecologic, Lung, Sarcoma	Oncology clinic	55	160 minutes	X		X		X	Low

© 2021 Feliciano JL et al. *JAMA Network Open.*

Hui, 2016[17]<br><br>RCT	24	Breast, Gastrointestinal, Genitourinary, Gynecologic, Lung, Hematologic, Other	Palliative care clinic	52.4	172 minutes	X		X		X	Low
Hui, 2017[16]<br><br>RCT	20	Breast, GI, GU, Gyn, Lung, Other	Palliative care clinic	55	6 minutes	X		X		X	Low
Pinna, 2015[19]<br><br>RCT: Crossover	13	Breast, Kidney, Lung, Stomach	Palliative care clinic	65.2	7 Days	X		X		X	Some concerns
**Opioids vs Anxiolytics**											
Navigante, 2010[25]<br><br>RCT	63	Breast, Head and neck, Lung, Other	Oncology clinic	Range 30-82	5 Days	X				X	Some concerns
**Opioids vs Anxiolytics vs Combination**											
Navigane, 2006[26]<br><br>RCT	101	Lung, Breast, Gynecologic, Sarcomas, Unknown primary, Colorectal, Other	Hospital	56.9-57.8	48 hours	X				X	High
**Opioids vs Corticosteroids vs Bronchodilators**											
Tian, 2016[29]<br><br>Retrospective cohort	343	Breast, Colorectal, Gastric, Lung, Other (not specified)	Hospital	53.1-54.2	60 minutes	X				X	Serious
**Opioids vs Opioids**											
Aabom, 2019[32]<br><br>RCT: Crossover	12	Lung and non-lung	Palliative care clinic	74.8	20 minutes	X					Low
Allard, 1999[28]	33	Breast, Lung/Pleura, Other (not specified)	Palliative care clinic	63.3	240 minutes	X				X	Some concerns

© 2021 Feliciano JL et al. *JAMA Network Open.*

RCT											
Bruera, 2005[27]<br><br>RCT: Crossover	12	Lung, Gastrointestinal, Other	Not reported	Median 58	2 days	X					High
Gamborg, 2013[24]<br><br>RCT	20	NR	Hospice (inpatient)	NR	1 hour	X				X	High
Hui, 2019[30]<br><br>RCT	30	Breast, Gastrointestinal, Genitourinary, Gynecological, Head and neck, Respiratory, Other (not specified)	Oncology, Palliative care clinic	52	NR	X				X	Low
Simon, 2016[23]<br><br>RCT: Crossover	10	Lung, Hematology, Breast, Ovary, Esophagus, Melanoma	Inpatient	58	60 minutes	X				X	High

© 2021 Feliciano JL et al. *JAMA Network Open.*

eTable 3. Summary of findings for the effects of pharmacological interventions on physiologic outcomes in patients with advanced cancer

Outcome	Comparison	Number of Studies Reporting Outcome (N)	Findings	Conclusion
**Blood pressure**				
	**Opioid vs. placebo**<br><br>Fentanyl vs. placebo (2)	**2 RCTs**<br><br>(N=44)	Pooled analysis:<br>• Diastolic, SMD: 0.243; 95% CI, -0.23 to 1.41<br>• Systolic, SMD: 0.478; 95% CI, -0.13 to 1.09	• There was no difference between opioids and placebo in the effect on blood pressure
	**Opioid vs. opioid**<br><br>High dose vs. low dose fentanyl	**1 RCTs**<br><br>(N=30)	• Diastolic, difference between beginning and end of walk, calculated SMD: 0.14; 95% CI, -0.54 to 0.81<br>• Systolic, difference between beginning and end of walk, calculated SMD: 0.17; 95% CI, -0.51 to 0.84	• There was no significant change in blood pressure in patients in either arm
**Heart rate**				
	**Opioids vs. placebo**<br><br>Fentanyl vs. placebo (2)<br>Hydromorphone (nebulized) vs. hydromorphone (Oral or SC) vs. placebo (nebulized) (1)	**3 RCTs**<br><br>(N=64)	Pooled analysis with Charles, 2008 et al.[12] saline vs. nebulized hydromorphone comparison:<br>• SMD: -0.14 (95% CI: -0.57 to 0.29),<br>• I-squared=0.0%, p=0.66<br><br>Pooled analysis with Charles, 2008 et al.[12] saline vs. systemic hydromorphone comparison:<br>• SMD: -0.03 (95% CI: -0.46 to 0.4)<br>• I-squared=0.0%, p=0.46	• There was no significant difference between opioids and placebo in the effect on heart rate.
	**Opioid vs. opioid**<br><br>sublingual vs. subcutaneous morphine (1)<br>High dose vs. low dose fentanyl (1)<br>Hydromorphone (nebulized) vs. hydromorphone (Oral or SC) vs. placebo (nebulized) (1)	**3 RCTs**<br><br>(N=75)	Pooled analysis:<br>• SMD: 0.11; 95% CI, -0.3 to 0.52<br>• I-squared=0.0%, p=0.79	• There was no significant difference between opioids in the effect on heart rate
**Oxygen saturation**				
	**Opioid vs. placebo**<br><br>Fentanyl vs. placebo (4)<br>Hydromorphone (nebulized) vs. hydromorphone (Oral or SC) vs. placebo (nebulized) (1)<br>Morphine vs. placebo	**6 RCTs**<br><br>(N=107)	Pooled analysis with Charles, 2008 et al.[12] saline vs. nebulized hydromorphone comparison:<br>• SMD: -0.07 (95% CI: -0.40 to 0.25),<br>• I-squared=0.0%, p=0.65<br><br>Pooled analysis with Charles, 2008 et al.[1] saline vs. systemic	• There was no difference between opioids and placebo in the effect on oxygen saturation.

	(1)		hydromorphone comparison:<br>• SMD: -0.13 (95% CI: -0.45 to 0.19)<br>• I-squared=0.0%, p=0.63	
	**Opioid vs. opioid**<br><br>Fentanyl vs. morphine (1)<br>High dose vs. low dose fentanyl (1)<br>Hydromorphone (nebulized) vs. hydromorphone (Oral or SC) vs. placebo (nebulized) (1)	3 RCTs<br><br>(N=65)	Pooled analysis:<br>• SMD: -0.03; 95% CI, -0.44 to 0.37<br>• I-squared=0.0%, p=0.60	• There was no difference in the effect on oxygen saturation between opioids.
	**Opioid vs. anxiolytics**<br><br>Oral morphine vs. oral midazolam (1)<br>Subcutaneous morphine vs. subcutaneous midazolam vs. combination (1)	2 RCTs<br><br>(N=164)	90 minutes (calculated SMD: 0.001, 95% CI, -0.49 to 0.5) or Day 5 (calculated SMD: -0.003, 95% CI, -0.5 to 0.49)<br><br>Second study reported no significant differences between groups. Unable to calculate SMD, no variability reported	• There was no difference in the effect on oxygen saturation for opioids compared to anxiolytics
**Respiratory rate**				
	Opioid vs. placebo<br><br>Fentanyl vs. placebo (3)<br>Hydromorphone (nebulized) vs. hydromorphone (Oral or subcutaneous) vs. placebo (nebulized) (1)<br>Morphine vs. placebo (**1**)	**5 RCTs**<br><br>(N=94)	Pooled analysis with Charles, 2008 et al.[12] saline vs. nebulized hydromorphone comparison:<br>• SMD: 0.11 (95% CI: -0.25 to 0.47),<br>• I-squared=0.0%, p=0.44<br><br>Pooled analysis with Charles, 2008 et al.[12] saline vs. systemic hydromorphone comparison:<br>• SMD: 0.05 (95% CI: -0.31 to 0.41)<br>• I-squared=1.0%, p=0.40	• There was no difference between opioids and placebo in the effect on respiratory rate.
	Opioid vs. opioid<br><br>Low dose vs. high dose opioid (drug unspecified) (1)<br>Morphine vs. fentanyl (**1**)<br>High dose vs. low dose fentanyl (1)<br>Hydromorphone (nebulized) vs. hydromorphone (Oral or SC) vs. placebo (nebulized) (1)	**4 RCTs**<br><br>(N=98)	Pooled analysis:<br>• SMD: -0.23 (95% CI, -0.63 to 0.18)<br>• I-squared=0.0%, p=0.91	• There was no difference between opioids in the effect on respiratory rate

SMD: standardized mean difference, RR: relative risk, MBGD: mean between group difference; vs= versus

© 2021 Feliciano JL et al. *JAMA Network Open.*

eTable 4. List of studies reporting harms and dropouts in studies of pharmacological interventions for breathlessness in patients with advanced cancer

Author, year	N	Central nervous system	Gastro-intestinal	Pruritus	Urinary retention	Dry mouth	Dropouts
**Opioids vs. Placebo**							
Hui, 2017[16]	20	X	X	X			X
Hui, 2016[17]	24	X	X				
Hui, 2014[18]	20	X	X	X			
Pinna, 2015[19]	13		X				
Charles, 2008[15]	20						X
**Anxiolytics vs. Placebo**							
Peoples, 2016[21]	379						X
Hardy, 2016[31]	73	X					
**Placebo vs. Corticosteroids vs. Placebo**							
Hui, 2016[22]	41	X	X				X
**Opioids vs. Opioids**							
Kawabata, 2013[33] [retro]	95	X	X	X	X		
Bruera, 2005[27]	12	X	X				
Hui, 2019[30]	30	X	X	X			
**Opioids vs. Anxiolytics**							
Navigante, 2010[25]	63	X	X	X		X	X
**Opioids vs. Anxiolytics vs. Combination**							
Navigane, 2006[26]	101	X	X			X	
**Opioids vs. Corticosteroids vs. Bronchodilators**							
Tian, 2016[29]	343	X	X			X	

N=sample size

© 2021 Feliciano JL et al. *JAMA Network Open.*

eTable 5. Rate of dropouts due to adverse effects of pharmacological interventions for breathlessness in patients with advanced cancer

Drug Class	Intervention	Dropouts due to adverse effects, n (%)
**Opioids**	Fentanyl[16]	1 (9.1%)
	Hydromorphone[15]	4 (16%)
	Morphine[25]	1 (3.2%)
**Anxiolytics**	Midazolam[25]	1 (3.2%)
	Buspirone[21]	10 (4.7%)
**Corticosteroids**	Dexamethasone[22]	1 (5.6%) – 1 (7.1%)

© 2021 Feliciano JL et al. *JAMA Network Open.*

eTable 6a. Risk of bias of randomized controlled trials that evaluate the effects of pharmacologic interventions

Author, year	Domain 1: Randomization process	Domain 2: Deviations intended interventions (effect of assignment to intervention)	Domain 2: Deviations intended interventions (effect of adhering to intervention)	Domain 3: Missing outcome data	Domain 4: Measurement of the outcome	Domain 5: Selection of the reported result	Final Assessment
Aabom, 2019[32]	Low risk	Low risk	NA	Low risk	Low risk	Low risk	Low risk
Allard, 1999[28]	Some concerns	Low risk	NA	Low risk	Low risk	Low risk	Some concerns
Bruera, 1993[20]	Some concerns	Low risk	NA	Low risk	High risk	Low risk	High risk
Bruera, 2005[27]	Some concerns	Low risk	NA	Some concerns	Low risk	Some concerns	High risk
Charles, 2008[15]	Some concerns	Low risk	NA	Low risk	Low risk	Some concerns	High risk
Gamborg, 2013[24]	Some concerns	Some concerns	NA	Low risk	Some concerns	Low risk	High risk
Hardy, 2016[31]	Low risk	Low risk	NA	Low risk	Low risk	Low risk	Low risk
Hui, 2014[18]	Low risk	Low risk	NA	Low risk	Low risk	Low risk	Low risk
Hui, 2016[17]	Low risk	Low risk	NA	Low risk	Low risk	Low risk	Low risk
Hui, 2016[22]	Low risk	Low risk	NA	Low risk	Low risk	Low risk	Low risk
Hui, 2017[16]	Low risk	Low risk	NA	Low risk	Low risk	Low risk	Low risk
Hui, 2019[30]	Low risk	Low risk	NA	Low risk	Low risk	Low risk	Low risk
Navigante, 2006[26]	Low risk	Some concerns	NA	Low risk	Some concerns	Some concerns	High risk
Navigante, 2010[25]	Low risk	Some concerns	NA	Low risk	Some concerns	Some concerns	Some concerns

© 2021 Feliciano JL et al. *JAMA Network Open.*

Peoples, 2016[21]	Low risk	Low risk	NA	Some concerns	Low risk	Low risk	Some concerns
Pinna, 2015[19]	Some concerns	Low risk	NA	Low risk	Low risk	Low risk	Some concerns
Simon, 2016[23]	Some concerns	Low risk	NA	Some concerns	High risk	High risk	High risk

© 2021 Feliciano JL et al. *JAMA Network Open*.

eTable 6b. Risk of bias of observational studies that evaluate the effects of pharmacologic interventions

Author, year	Domain 1: Confounding	Domain 2: Patient Selection	Domain 3: Classifying Interventions	Domain 4: Deviations from intended interventions	Domain 5: Missing data	Domain 6: Measurement of outcomes	Domain 7: Selection of reported results	Overall Assessment
Kawabata, 2013[33]	Critical	Serious	Low	No information	Moderate	Moderate	Serious	Critical
Tian, 2016[29]	Serious	Moderate	Low	Moderate	Moderate	Moderate	Serious	Serious

© 2021 Feliciano JL et al. *JAMA Network Open*.

eTable 7. Strength of evidence of studies that evaluate the effects of pharmacologic interventions

Key Outcome	Intervention	Number of studies (participants)	Study limitations	Directness	Consistency	Precision	Reporting bias	Strength of evidence
Breathlessness	Opioids vs Placebo	6 RCT (107 participants)	Low	Direct	Consistent	Precise	Undetected	Moderate
Breathlessness	Anxiolytics vs Placebo	2 RCT (452 participants)	Medium	Direct	Consistent	Imprecise	Undetected	Low
Breathlessness	Corticosteroids vs Placebo	1 RCT (41 participants)	Low	Direct	Unknown	Imprecise	Suspected	Insufficient
Breathlessness	Opioids vs Opioids	7 RCT (142 participants)	High	Direct	Consistent	Imprecise	Suspected	Low
Breathlessness	Opioids vs Anxiolytics	2 RCT (164 participants)	Medium	Direct	Inconsistent	Imprecise	Suspected	Low
Breathlessness	Opioids vs Corticosteroids vs Bronchodilators	1 retrospective cohort (343 participants)	High	Direct	Unknown	Imprecise	Suspected	Insufficient
Anxiety	Anxiolytics vs Placebo	2 RCT (452 participants)	Medium	Direct	Consistent	Precise	Undetected	Low
Health-related quality of life	Corticosteroids vs Placebo	1 RCT (41 participants)	Low	Direct	Unknown	Imprecise	Suspected	Insufficient
Exercise capacity	Opioids vs Placebo	4 RCT (77 participants)	Low	Direct	Consistent	Precise	Undetected	Moderate

RCT=randomized clinical trial

© 2021 Feliciano JL et al. *JAMA Network Open.*

eFigure 1. Analytic Framework for Evaluating Interventions for Breathlessness in Patients with Advanced Cancer

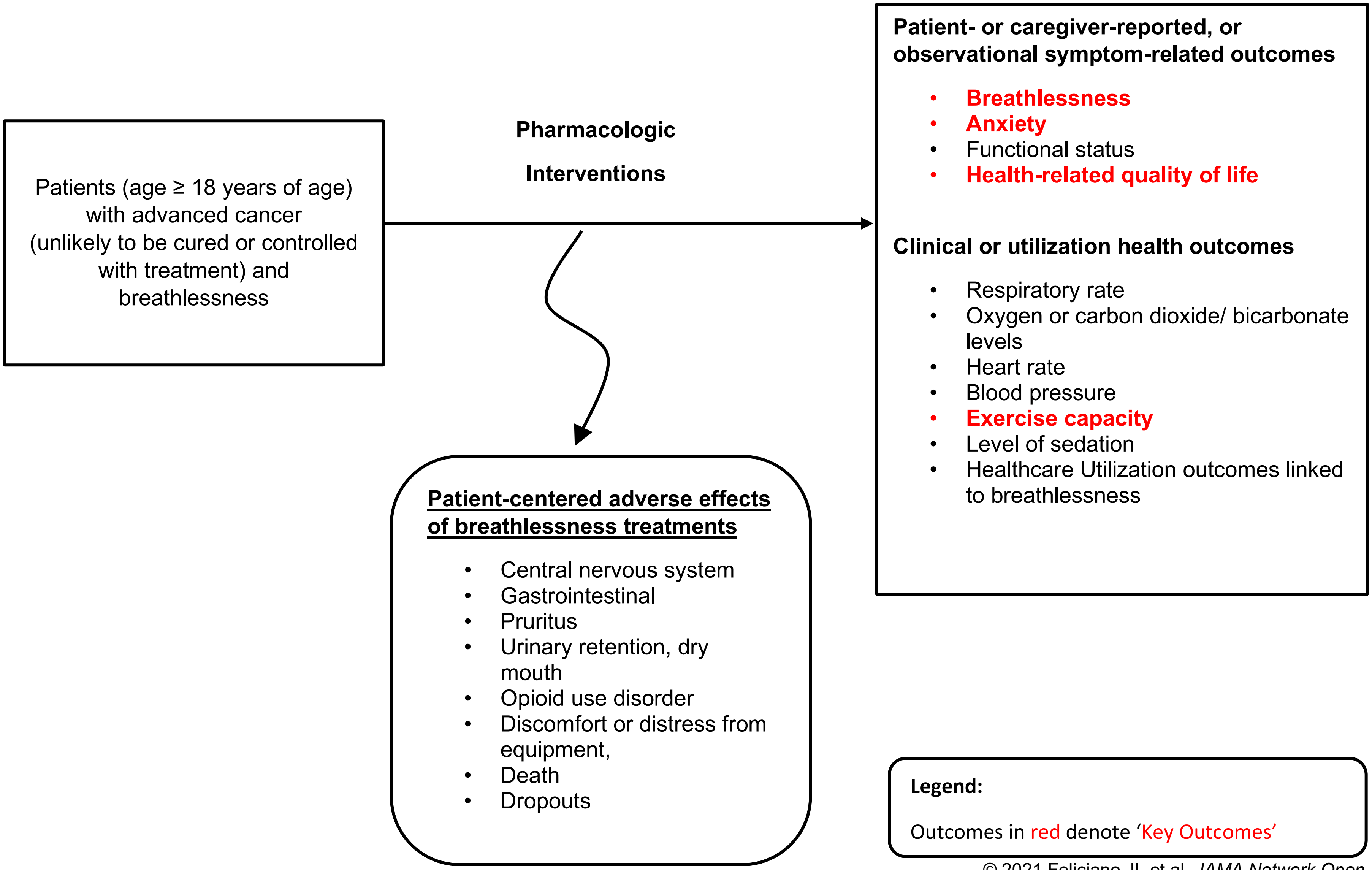

© 2021 Feliciano JL et al. *JAMA Network Open*.

eFigure 2. Study Search and Preferred Reporting Items for Systematic Reviews and Meta-analyses Flowchart

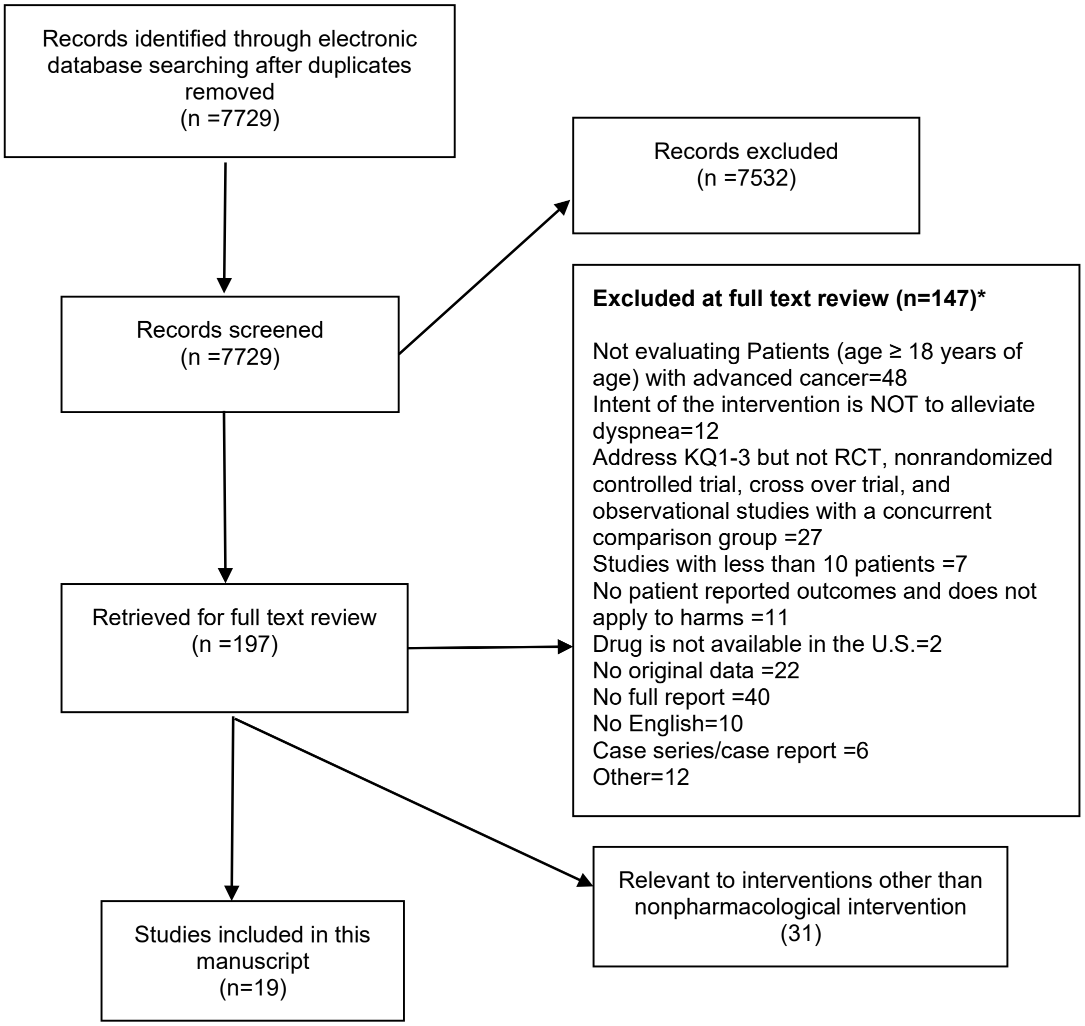

* Total exceeds the number of citations in the exclusion box, because citations could be excluded for more than one reason

© 2021 Feliciano JL et al. *JAMA Network Open.*

eFigure 3. Meta-analysis of the effects on exercise capacity measures in randomized controlled trials comparing opioids with placebo in patients with advanced cancer

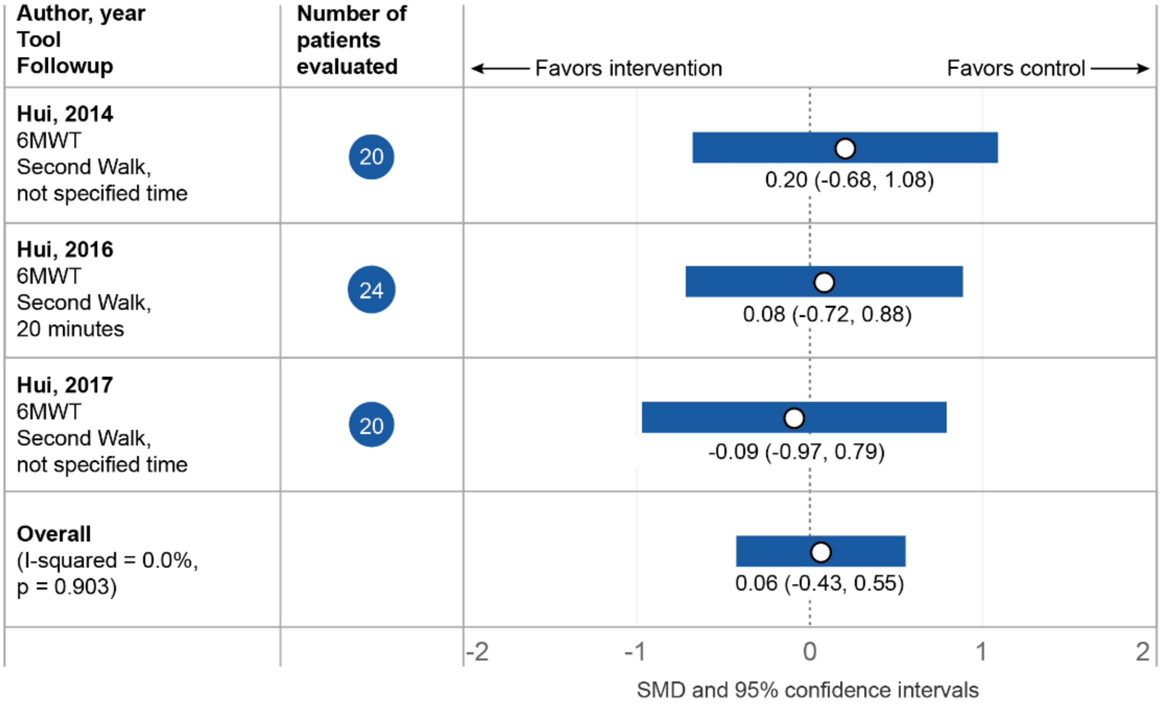

Blue dot size=corresponds to study size, Blue diamond=the result when all the individual studies are combined and averaged
Length of the bar=corresponds to range of confidence interval.

© 2021 Feliciano JL et al. *JAMA Network Open.*

eFigure 4. Meta-analysis of the effects of placebo vs opioids on blood pressure in patients with advanced cancer in inpatient hospice or palliative care units

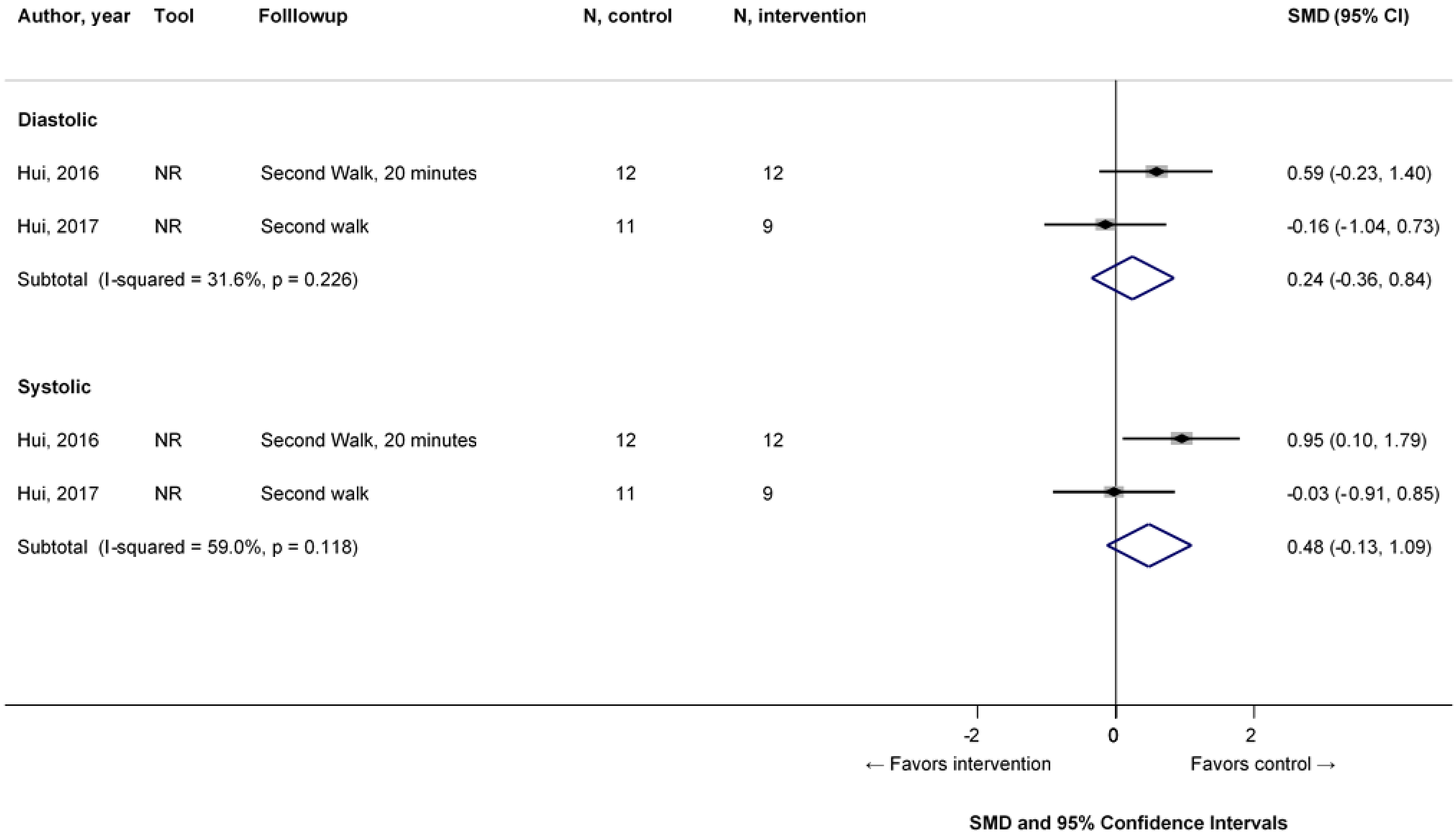

CI=confidence interval; N=sample size; NR=not reported; SMD=standardized mean difference

© 2021 Feliciano JL et al. *JAMA Network Open*.

eFigure 5. **Meta-analysis of the effects of placebo vs opioids on heart rate in patients with advanced cancer in inpatient hospice or palliative care units (Charles, 2008 et al.[15] saline vs nebulized hydromorphone comparison)**

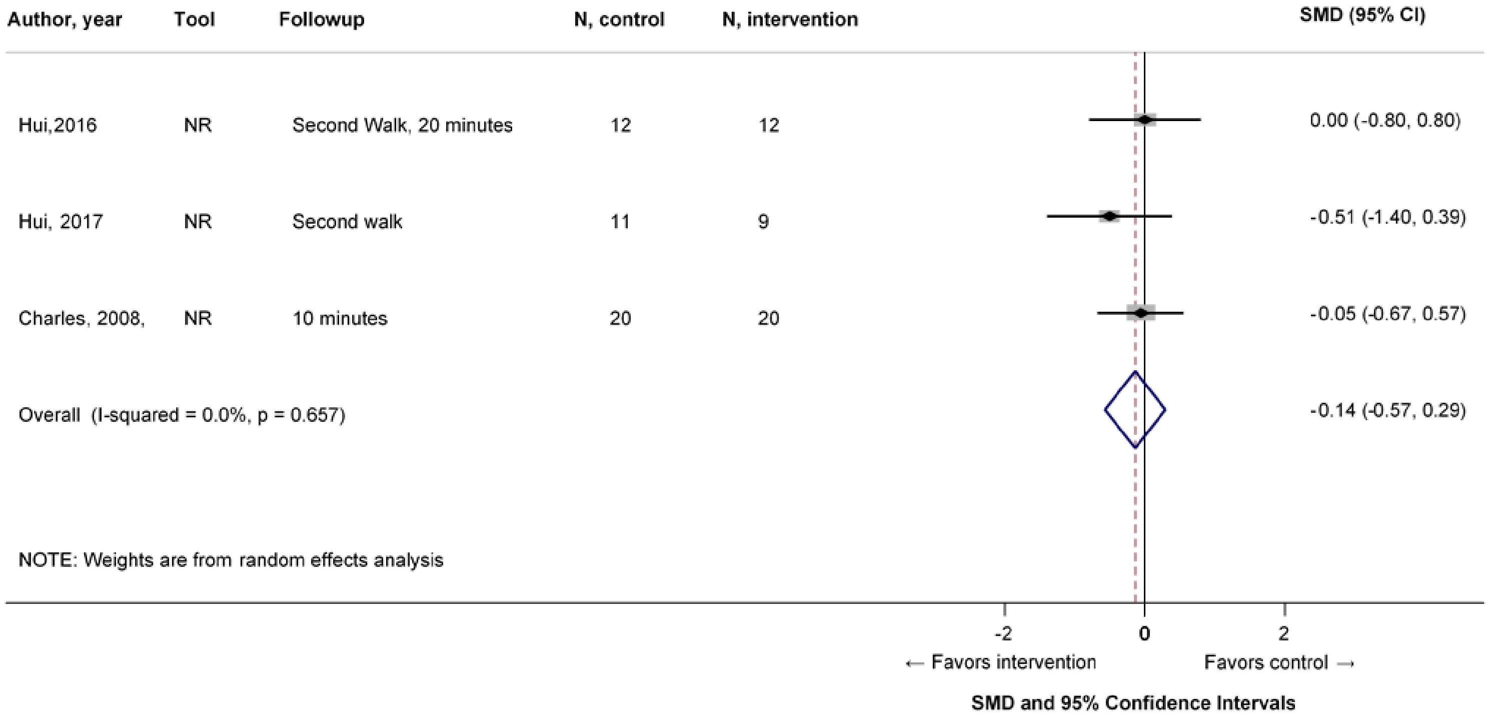

CI=confidence interval; N=sample size; NR=not reported; SMD=standardized mean difference

© 2021 Feliciano JL et al. *JAMA Network Open.*

eFigure 6. Meta-analysis of the effects of placebo vs opioids on heart rate in patients with advanced cancer in inpatient hospice or palliative care units (Charles, 2008 et al.[15] saline vs systemic hydromorphone comparison)

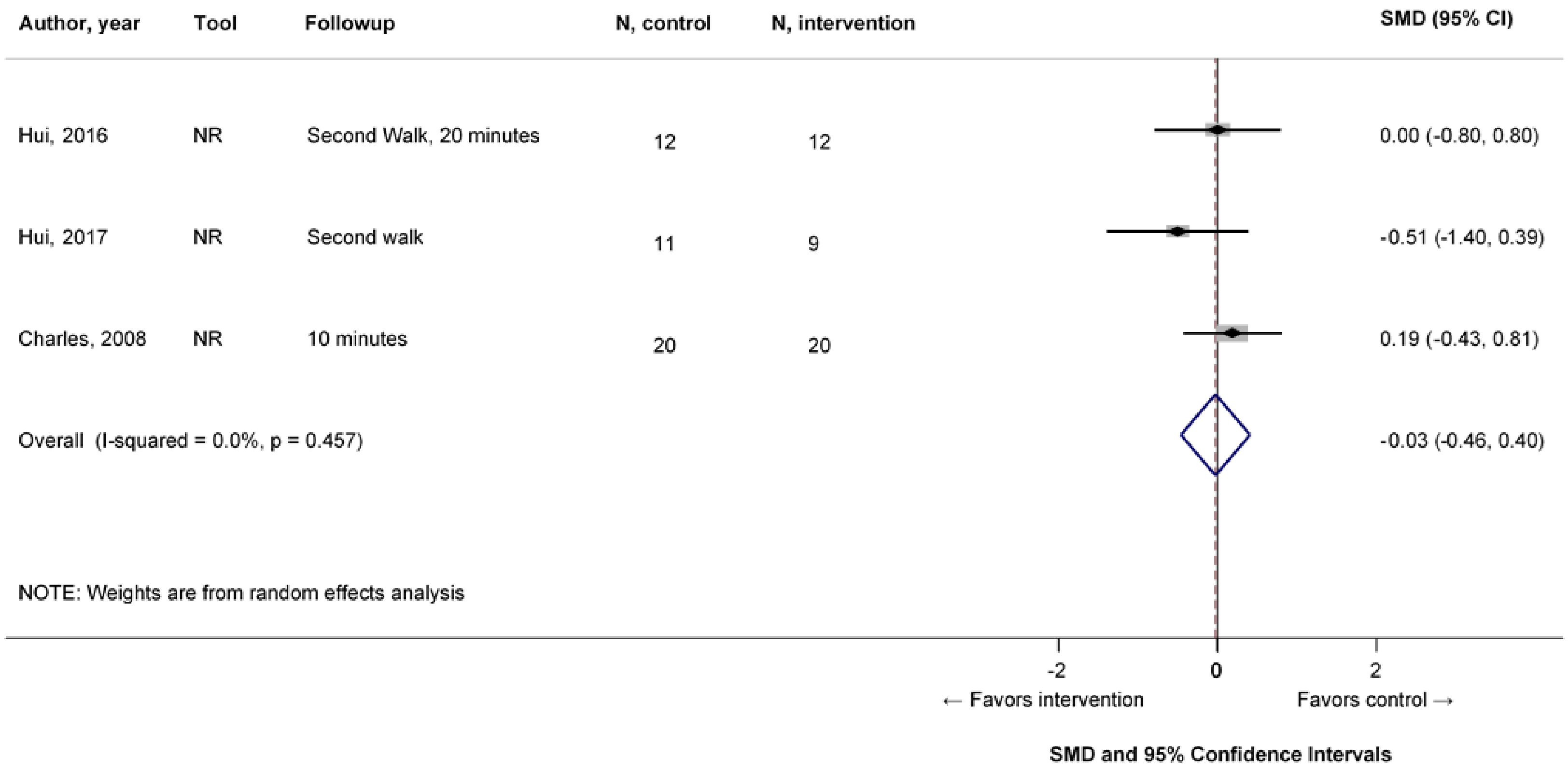

CI=confidence interval; N=sample size; NR=not reported; SMD=standardized mean difference

© 2021 Feliciano JL et al. *JAMA Network Open*.

eFigure 7. **Meta-analysis of the effects of opioids vs opioids on heart rate in patients with advanced cancer in inpatient hospice or palliative care units**

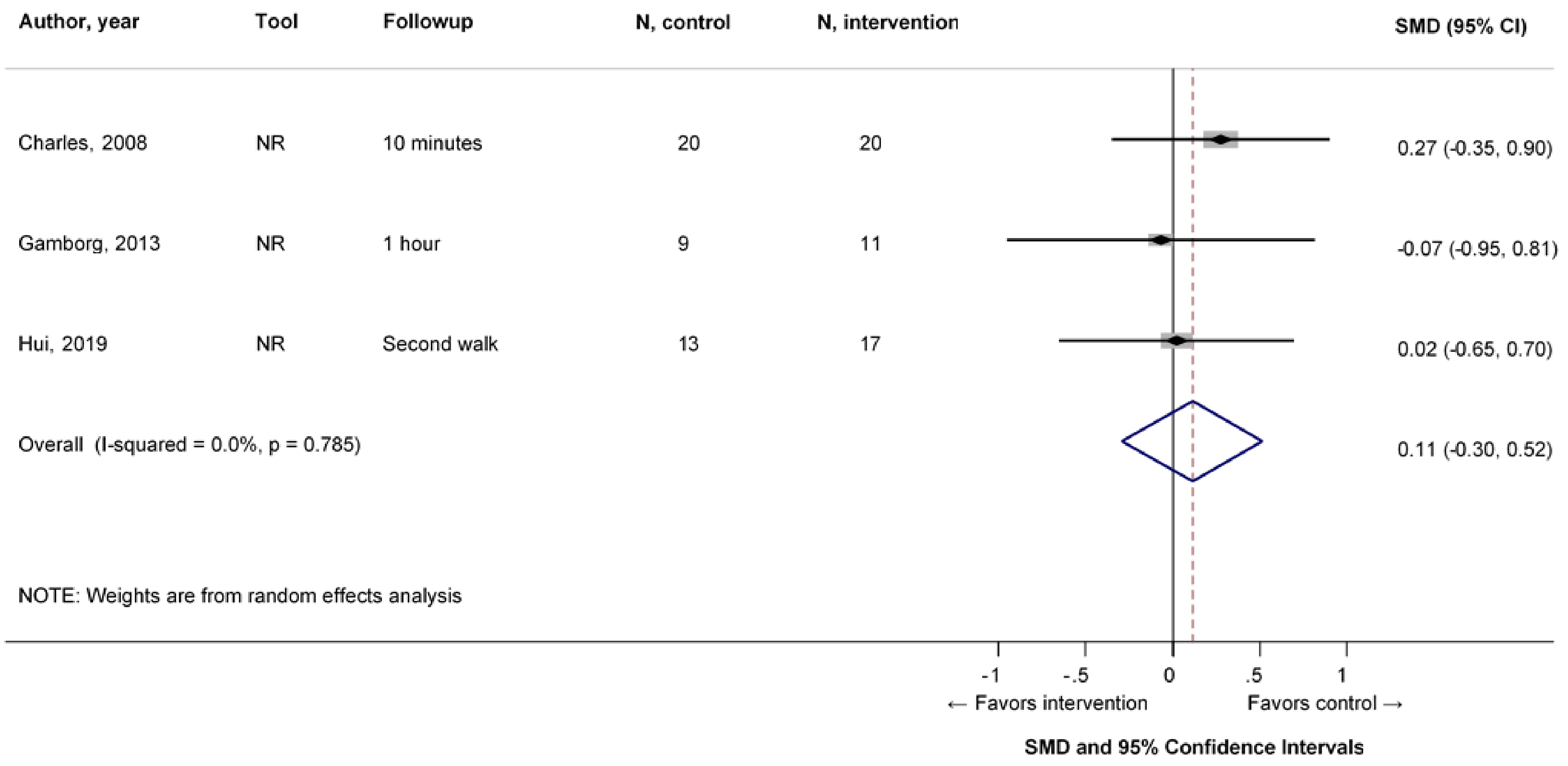

CI=confidence interval; N=sample size; NR=not reported; SMD=standardized mean difference

© 2021 Feliciano JL et al. *JAMA Network Open*.

eFigure 8. Meta-analysis of the effects of placebo vs opioids on oxygen saturation in patients with advanced cancer in inpatient hospice or palliative care units (Charles, 2008 et al.[15] saline vs nebulized hydromorphone comparison)

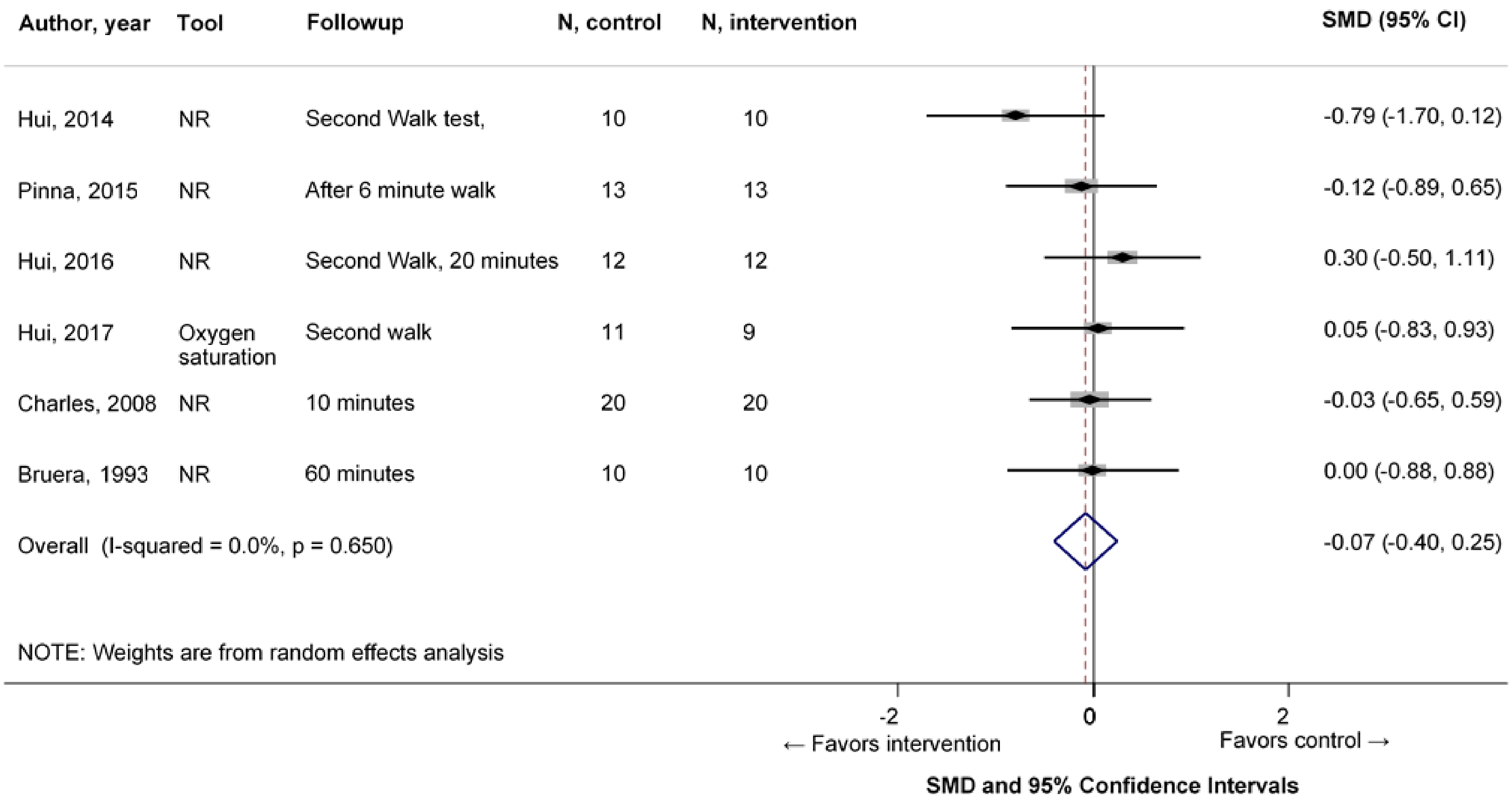

CI=confidence interval; N=sample size; NR=not reported; SMD=standardized mean difference

© 2021 Feliciano JL et al. *JAMA Network Open*.

eFigure 9. Meta-analysis of the effects of placebo vs opioids on oxygen saturation in patients with advanced cancer in inpatient hospice or palliative care units (Charles, 2008 et al.[15] saline vs systemic hydromorphone comparison)

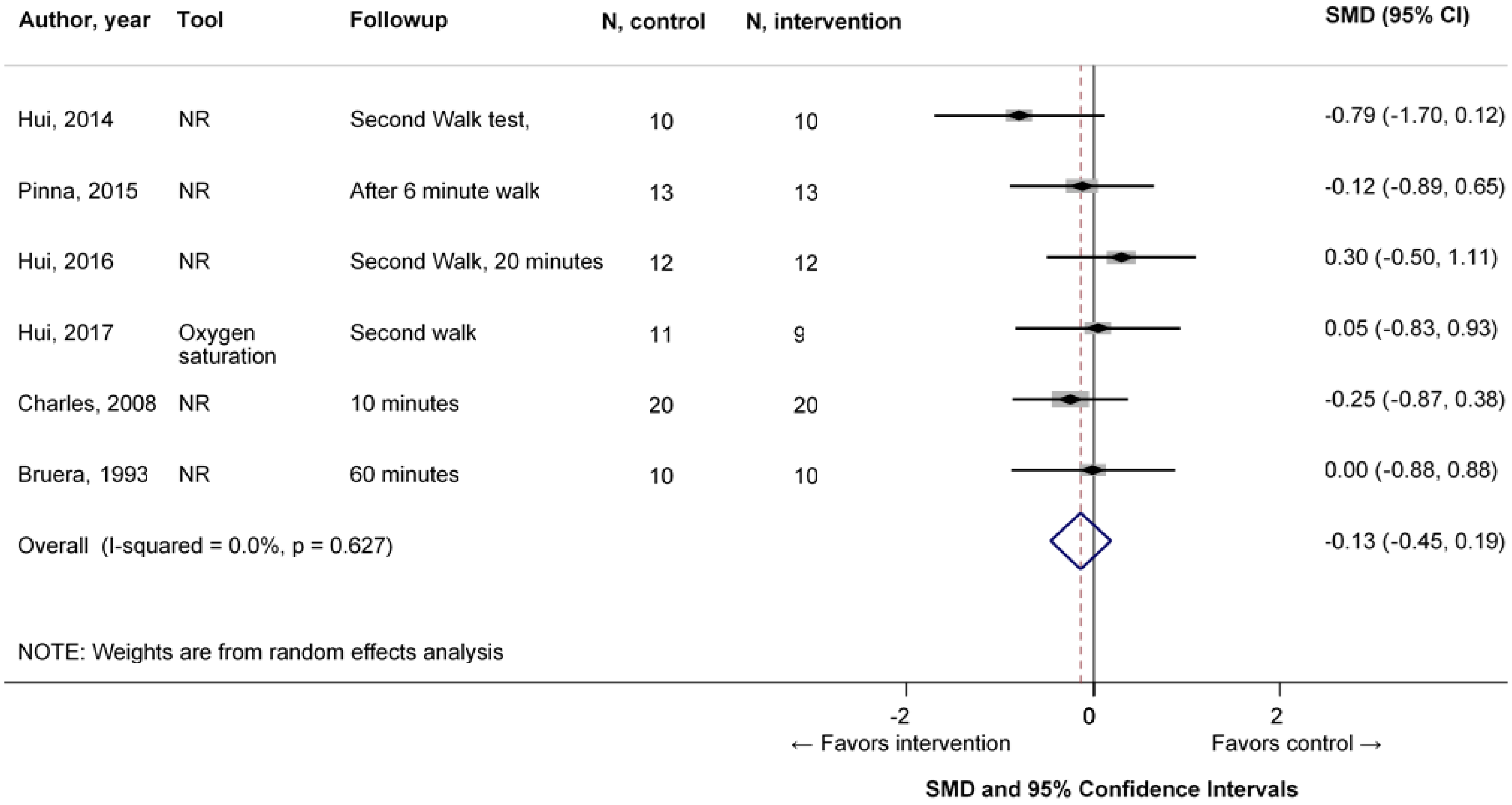

CI=confidence interval; N=sample size; NR=not reported; SMD=standardized mean difference

© 2021 Feliciano JL et al. *JAMA Network Open*.

eFigure 10. Meta-analysis of the effects of opioids vs opioids on oxygen saturation in patients with advanced cancer in inpatient hospice or palliative care units

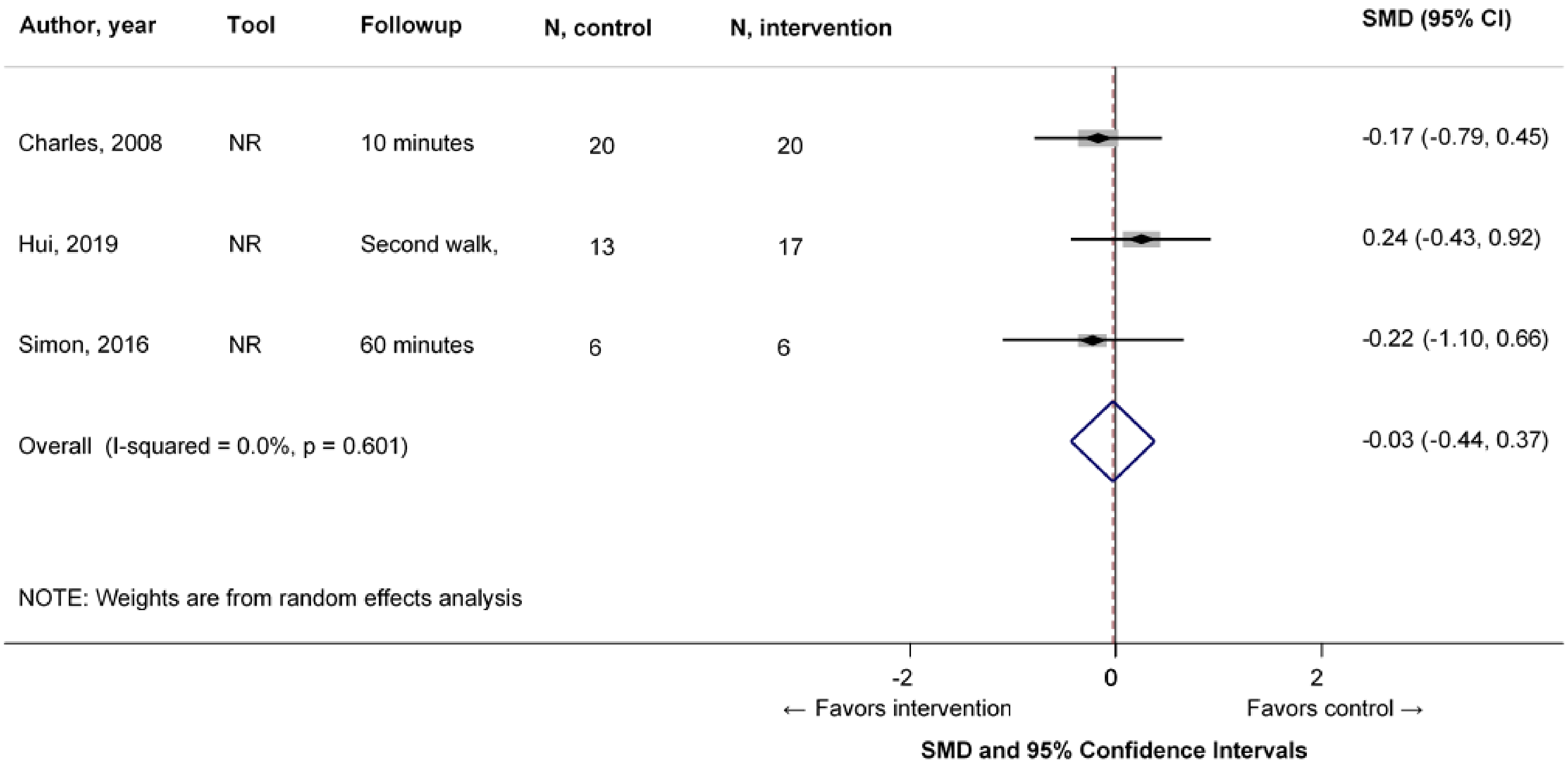

CI=confidence interval; N=sample size; NR=not reported; SMD=standardized mean difference

© 2021 Feliciano JL et al. *JAMA Network Open*.

eFigure 11. Meta-analysis of the effects of placebo vs opioids on respiratory rates in patients with advanced cancer in inpatient hospice or palliative care units (Charles, 2008 et al.[15] saline vs nebulized hydromorphone comparison)

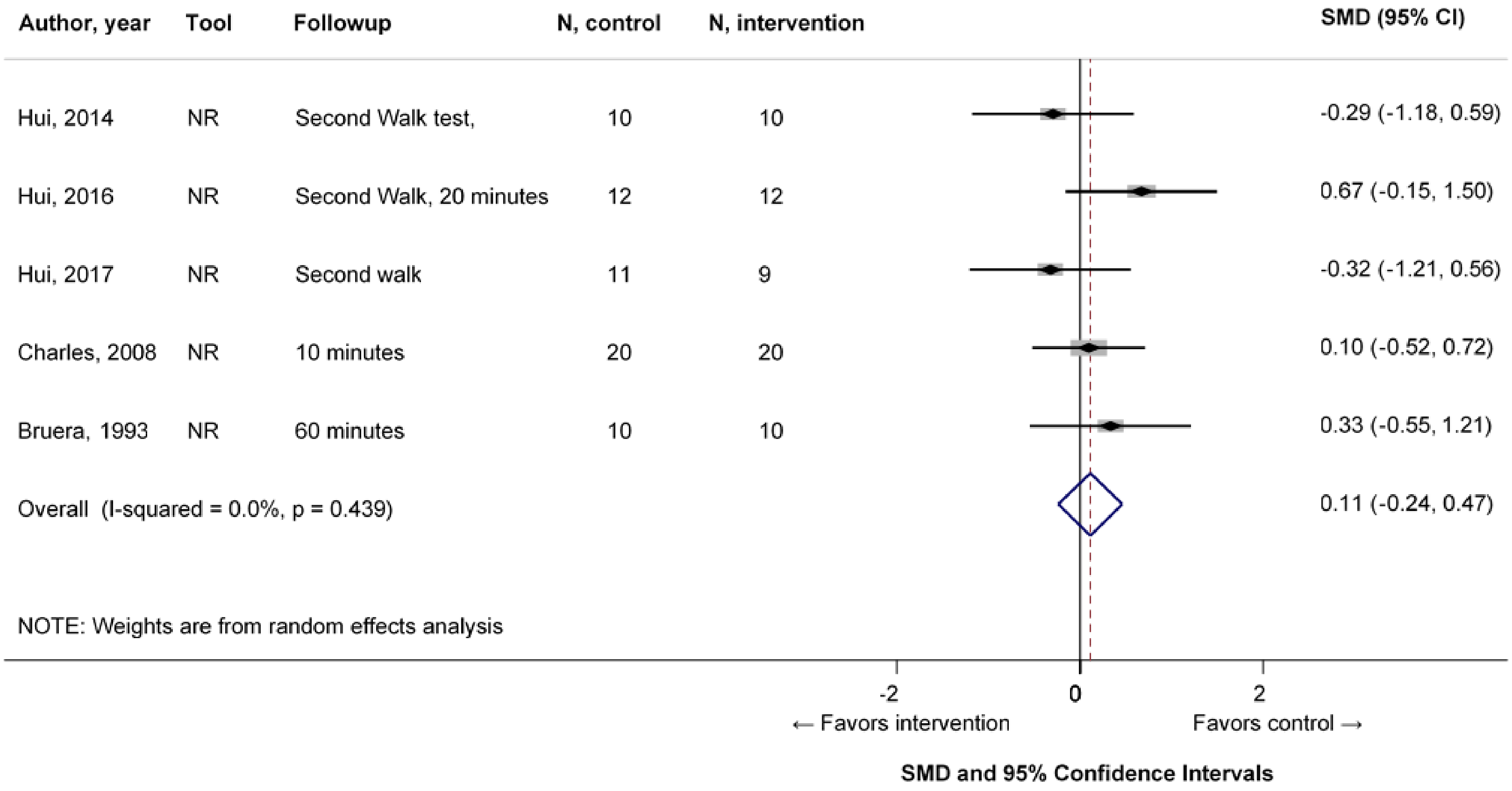

CI=confidence interval; N=sample size; NR=not reported; SMD=standardized mean difference

© 2021 Feliciano JL et al. *JAMA Network Open*.

eFigure 12. Meta-analysis of the effects of placebo vs opioids on respiratory rates in patients with advanced cancer in inpatient hospice or palliative care units (Charles, 2008 et al.[15] saline vs systemic hydromorphone comparison)

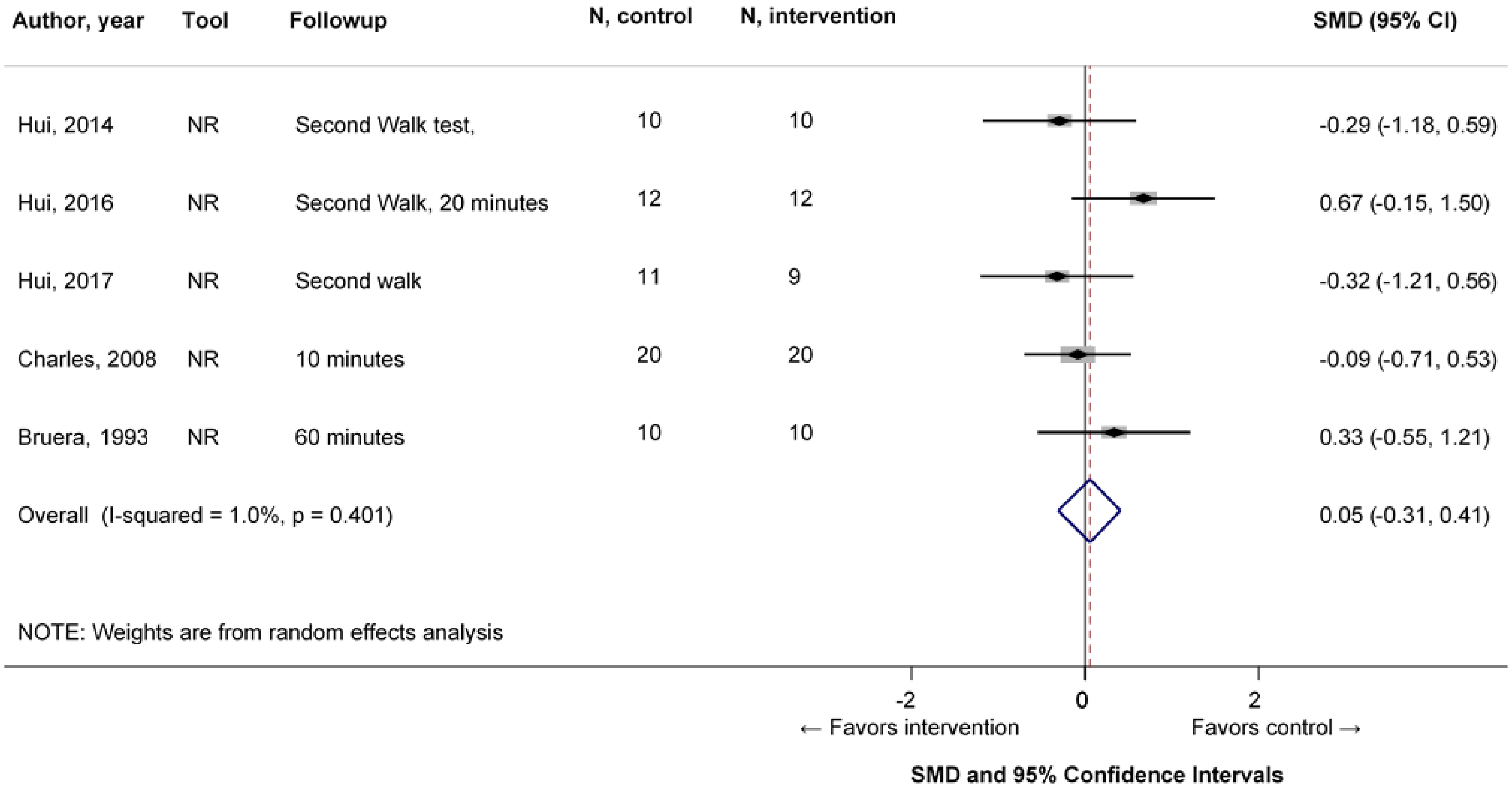

CI=confidence interval; N=sample size; NR=not reported; SMD=standardized mean difference

© 2021 Feliciano JL et al. *JAMA Network Open*.

eFigure 13. Meta-analysis of the effects of opioids vs opioids on respiratory rates in patients with advanced cancer in inpatient hospice or palliative care units

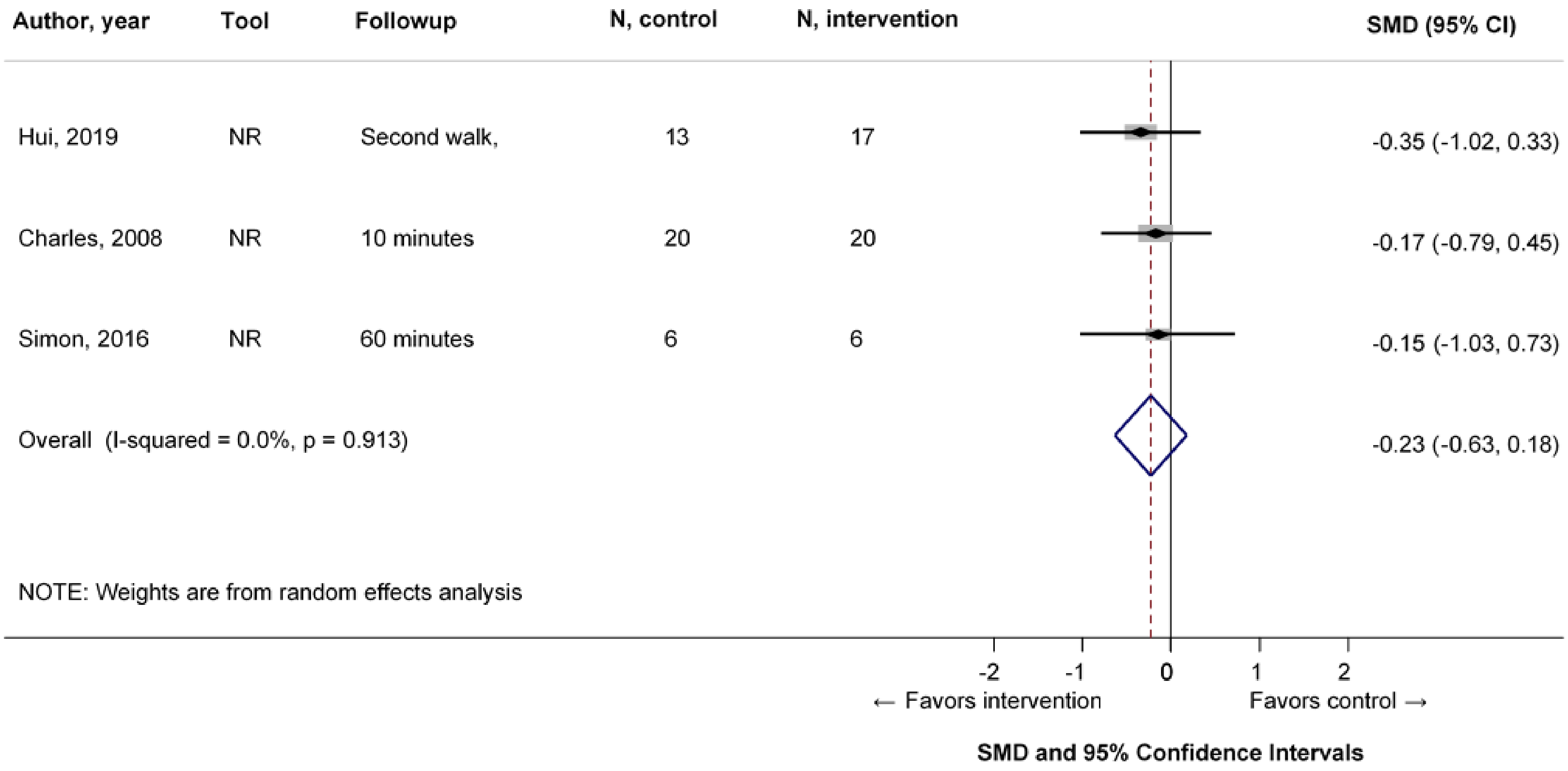

CI=confidence interval; N=sample size; NR=not reported; SMD=standardized mean difference

© 2021 Feliciano JL et al. *JAMA Network Open*.

Meta-analysis of harms of pharmacological interventions

eFigure 14. Meta-analysis of the effects of placebo vs opioids on dizziness outcomes in patients with advanced cancer in inpatient hospice or palliative care units

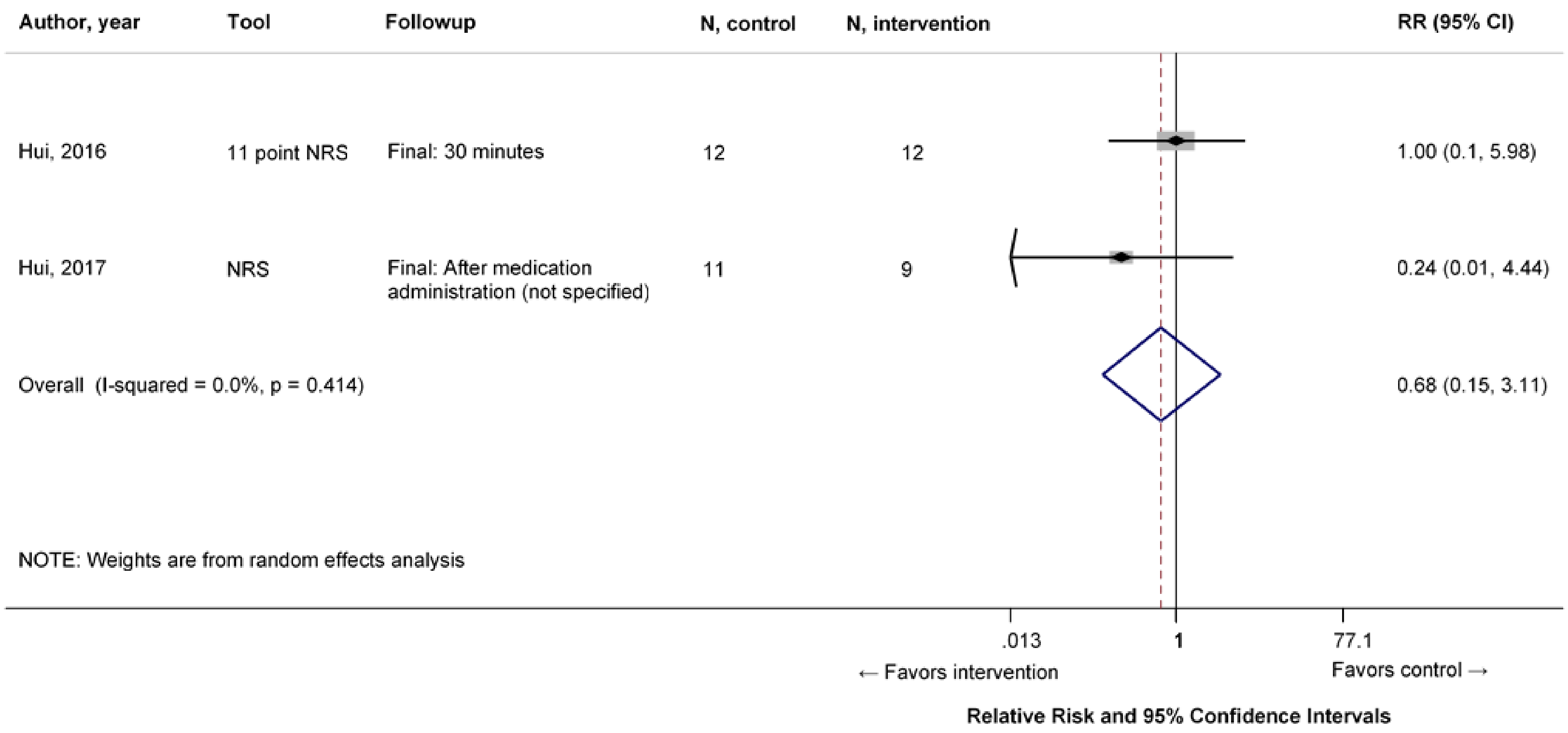

CI=confidence interval; N=sample size; NR=not reported; NRS=Numerical Rating Scale; RR=relative risk

© 2021 Feliciano JL et al. *JAMA Network Open*.

eFigure 15. Meta-analysis of the effects of placebo vs opioids on drowsiness outcomes in patients with advanced cancer in inpatient hospice or palliative care units

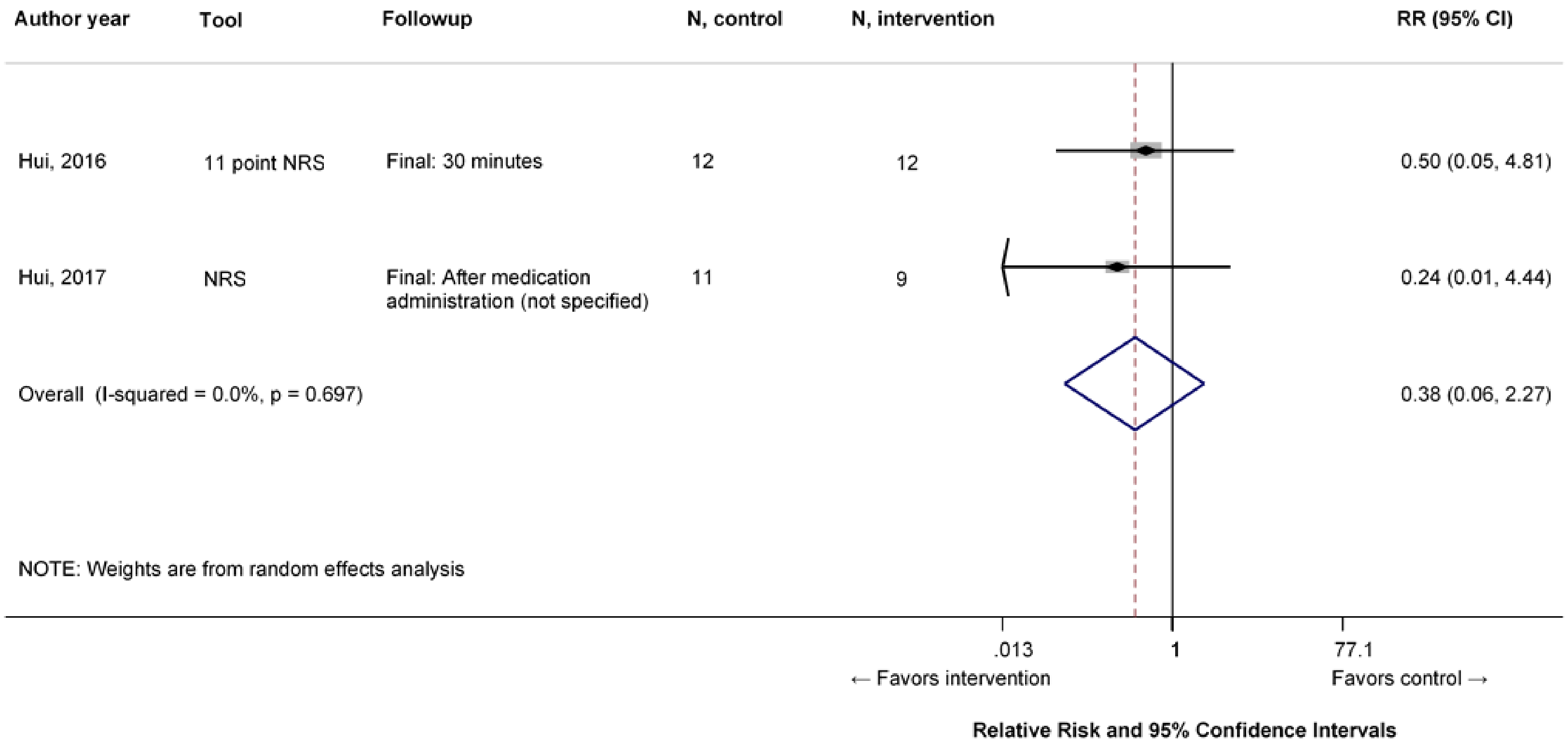

CI=confidence interval; N=sample size; NR=not reported; NRS=Numerical Rating Scale; RR=relative risk

© 2021 Feliciano JL et al. *JAMA Network Open*.

eFigure 16. Meta-analysis of the effects of placebo vs opioids on fatigue outcomes in patients with advanced cancer in inpatient hospice or palliative care units

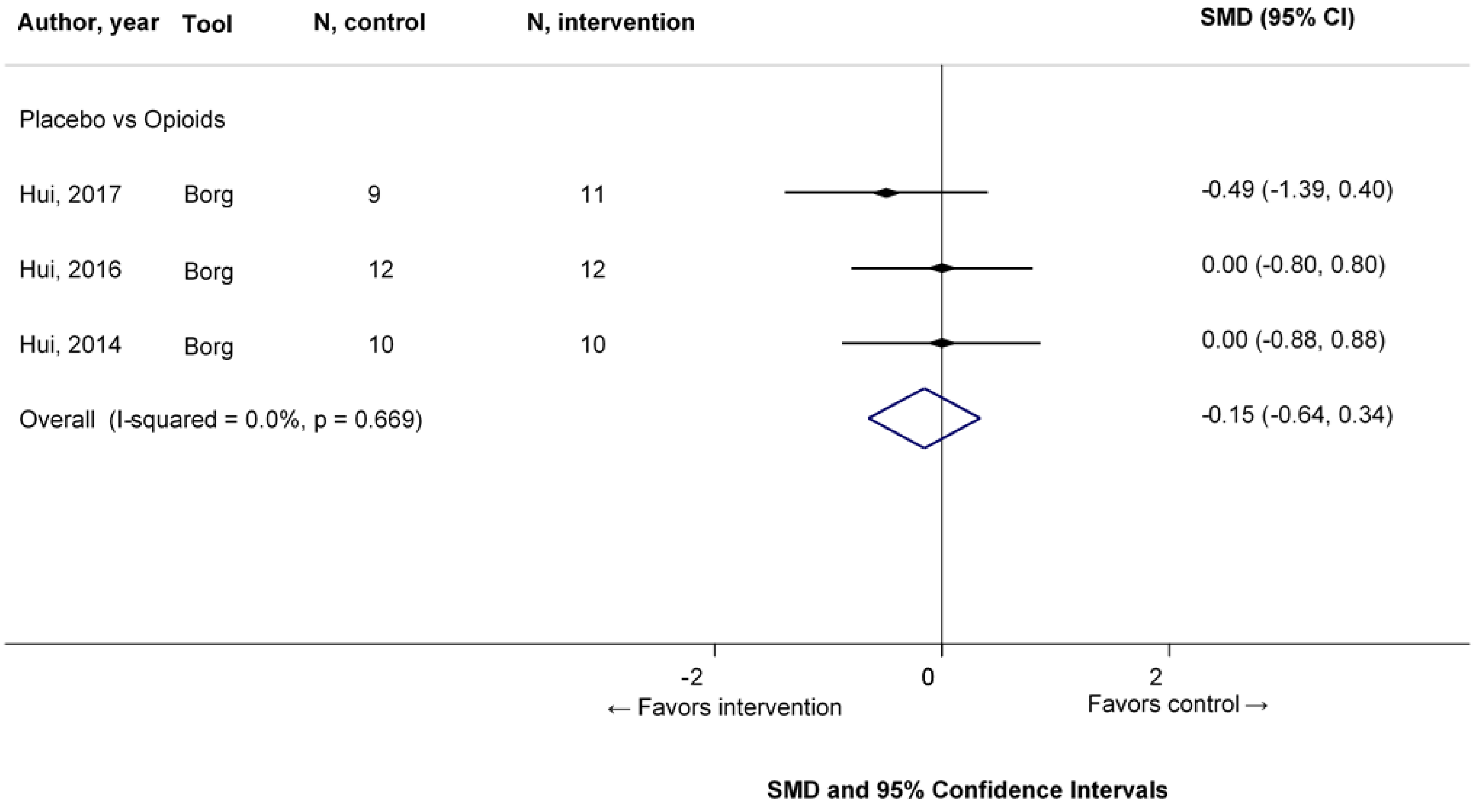

CI=confidence interval; N=sample size; NR=not reported; SMD=standardized mean difference

© 2021 Feliciano JL et al. *JAMA Network Open.*
